# Carbon starvation enhances microbiologically influenced corrosion in marine offshore infrastructures

**DOI:** 10.3389/fmicb.2026.1908395

**Published:** 2026-08-31

**Authors:** Sara Taghavi Kalajahi, Jan Lisec, Elyas Ghafoori, Maria Salta, Torben Lund Skovhus, Andrea Koerdt

**Affiliations:** 1 Department of Materials and Environment, Federal Institute for Materials Research and Testing (BAM), Berlin, Germany; 2 Department of Analytical Chemistry, Federal Institute for Materials Research and Testing (BAM), Berlin, Germany; 3 Institute for Steel Construction, ForWind, Leibniz University Hannover (LUH), Hannover, Germany; 4 Endures B.V. (Department: Biofilms, Biofouling and Fouling Control), Den Helder, Netherlands; 5 School of the Environment and Life Sciences, University of Portsmouth, Portsmouth, United Kingdom; 6 Research Centre for Built Environment, Climate and Water Technology, VIA University College, Horsens, Denmark

**Keywords:** carbon starvation, microbiologically influenced corrosion (MIC), offshore wind foundation, pitting corrosion, sulfate-reducing bacteria

## Abstract

**Introduction:**

In offshore wind foundations, microbiologically influenced corrosion (MIC) is a critical concern in the mud zone, where steel is embedded in sediment characterized by limited oxygen availability and pronounced redox gradients. In marine sediments, the limited availability of labile substrates can influence microbial metabolic strategies, potentially promoting alternative electron-transfer pathways. Here, we performed an exploratory mechanistic study to investigate how carbon availability influences microbial community structure and corrosion behavior in multispecies biofilms relevant to offshore infrastructure.

**Methods:**

Sediment from the North Sea was used as inoculum, and steel coupons were exposed under blank (no added carbon), lactate, and yeast extract conditions. Corrosion was assessed by weight loss and 3D profiling, while microbial communities were analyzed using 16S rRNA gene sequencing.

**Results and discussion:**

The findings illustrated that nutrient regime strongly shaped microbial and chemical conditions. Yeast extract produced the highest total sulfide concentrations (9 mM), but corrosion severity did not scale with bulk sulfide accumulation. The highest corrosion rate occurred under carbon-limited conditions (0.55 mm yr^−1^), compared with 0.122 and 0.072 mm yr^−1^ for lactate and yeast extract, respectively. Pitting rate was also most pronounced under carbon-limited conditions (0.65 mm yr^−1^). These findings suggest that the test conditions for offshore foundation materials should include low-labile-carbon scenarios to better reflect sediment zones and highlight the need for further mechanistic investigation.

## 1 Introduction

Marine corrosion remains one of the main degradation processes affecting metallic materials exposed to seawater, driven by the coupled effects of salinity, dissolved oxygen, moisture, hydrodynamics, and biological activity (Kotu et al., 2025). Corrosion severity varies across marine exposure zones (including atmospheric, splash, tidal, submerged, and mud zones) each characterized by distinct physicochemical and microbiological conditions that govern corrosion mechanisms and damage morphology. Among marine infrastructures, offshore wind structures have emerged as critical assets in global decarbonization strategies. Their structural reliability depends fundamentally on the long-term durability of steel foundations operating under aggressive mechanical and environmental loading.

Offshore wind foundations (such as monopiles) experience cyclic loading from wind, waves, and rotor operation, resulting in significant fatigue stresses that frequently control structural design life. Superimposed on these mechanical loads, corrosion [particularly microbiologically influenced corrosion (MIC)] causes material loss, localized corrosion (such as pitting), and stress concentration, thereby accelerating fatigue crack initiation and propagation (Shojai et al., 2025). The most critical loading region frequently occurs just below the seabed within the so-called mud zone, where bending moments are highest (Momber, 2024). Yet this region remains among the least accessible sections of offshore foundations for inspection and maintenance. As a result, uncertainties in corrosion rates, mechanisms, and spatial variability within sediments are commonly addressed through conservative design assumptions, increasing costs while still leaving residual risk.

The mud zone represents a uniquely complex corrosion environment. Steel in this region is in direct contact with marine sediment, where oxygen penetration is limited, creating pronounced redox gradients. Such conditions favor anaerobic microbial activity and MIC. Cathodic protection may also be less effective in sediments due to reduced ionic conductivity and shielding effects, further increasing susceptibility to localized corrosion (Momber, 2024). Consequently, understanding the MIC mechanisms in near seabed region is essential for realistic service-life prediction of offshore monopiles. MIC encompasses a spectrum of processes by which microorganisms modify corrosion behavior, either indirectly through metabolic activity called metabolite-mediated MIC (M-MIC) or directly through electrochemical interactions with metal surfaces entitled extracellular electron transfer (EET)-mediated MIC (EET-MIC) (Xu et al., 2023; Tüccar et al., 2025). In natural multispecies biofilms, these mechanisms rarely operate independently; rather, they occur simultaneously within spatially structured communities where redox stratification, metabolite retention, and microbe–surface interactions collectively govern corrosion processes (Xu et al., 2023). Importantly, MIC mechanisms arise from the interplay between microbial community structure and a broad range of environmental factors, with carbon availability being one (though not the only) parameter influencing metabolic pathways and electron-transfer processes (Yuxuan et al., 2022).

Traditionally, MIC has primarily been interpreted as a metabolite-driven process, in which microorganisms utilize available organic substrates and generate corrosive metabolic products such as hydrogen sulfide, organic acids, or differential aeration conditions that accelerate metal degradation. Within this framework, environments with limited bioavailable organic carbon are often assumed to pose a lower MIC threat because reduced nutrient availability is expected to constrain microbial activity and metabolite production. This assumption is particularly relevant for marine sediments surrounding offshore foundations, where a large fraction of the organic matter is refractory and only a limited portion is readily accessible to microorganisms (Jørgensen and Marshall, 2016). However, growing evidence indicates that low nutrient availability does not necessarily correspond to low corrosion severity. Pure-culture laboratory studies have demonstrated that some microorganisms can shift toward utilizing metallic iron or other energetic metals as alternative electron donors under carbon-limited conditions, potentially accelerating corrosion through EET-associated processes (Xu and Gu, 2014; Cui et al., 2025; Li et al., 2021). For example, carbon-starved *Desulfovibrio vulgaris* biofilms have been shown to significantly accelerate nickel corrosion by shifting toward metal-dependent electron acquisition pathways (Pu et al., 2023). These findings suggest that carbon limitation can fundamentally alter microbial survival strategies and intensify microbe–metal interactions. However, it remains insufficiently understood whether low bioavailable carbon in marine sediments hosting complex natural microbial consortia necessarily results in lower MIC threat for offshore wind foundations. Addressing this question is particularly important for offshore wind foundations in the mud zone, where limited nutrient availability, restricted mass transport, and complex biofilm development may strongly influence localized corrosion processes and long-term structural degradation.

In this study, sediment samples collected from the North Sea were used as inoculum to investigate the role of carbon availability in shaping microbial community composition and corrosion behavior. Three conditions were established: (i) unamended sediment representing baseline carbon-limited conditions, (ii) amendment with lactate as a defined low-molecular-weight electron donor, and (iii) amendment with yeast extract as a complex organic substrate. By coupling corrosion rate measurements with microbial community analysis, we evaluated how carbon limitation and nutrient amendment influenced sediment microbial structure and corrosion response. The results provide insight into potential starvation-associated MIC processes in near-bed marine environments and contribute to a better understanding of MIC threats affecting offshore monopile foundations. This approach allowed us to assess treatment-dependent patterns in corrosion, sulfide production, and microbial community structure, while providing a basis for future studies to examine the specific corrosion mechanisms in more detail.

## 2 Materials and methods

### 2.1 Materials

Seawater and sediment samples were collected from the North Sea by Endures BV Company (the Netherlands). The seawater samples were obtained near the Endures facility, close to the shoreline (Nieuwe haven, Den Helder, the Netherlands), while the sediment samples were collected from a site near an offshore wind park at approximately 5 m below the seafloor surface. Sediment samples were collected using a hollow liner to retrieve vertical sediment cores. The cores were sealed and transported under anaerobic conditions. Endures subsequently subsampled the material and supplied 1 L of sediment to our laboratory in airtight bottles under a nitrogen headspace to maintain anoxic conditions. The exact *in situ* temperature during sampling was not recorded; however, sampling took place in September, when typical North Sea temperatures range between approximately 15–20 °C. Prior to experimental use, samples were stored at 4 °C. All laboratory experiments were conducted under a fume hood, and the temperature was 21 ± 0.5 °C, which is within the upper range of natural environmental conditions. The seawater used in this study was characterized by a chloride concentration of 15,824 mg L^−1^, nitrate of 1.96 mg L^−1^, and sulfate of 2,135.8 mg L^−1^. Electrical conductivity was 4,306 μS cm^−1^, and the initial pH was 8.26.

Carbon steel coupons were used as metal specimens in this study (0.453 ± 0.02 g). According to manufacturer specifications (Goodfellow GmbH, Hamburg, Germany), the nominal chemical composition was: Fe 99.5%, Mn 0.3%, Si 0.1%, C < 0.08%, P < 0.04%, and S < 0.05%. The material was supplied as larger sheets and machined into coupons with dimensions of 0.8 × 0.8 × 0.1 cm. While the chemical composition of the coupons represents a relatively pure low-carbon steel, offshore wind monopiles are typically manufactured from structural steels (e.g., S355) containing higher levels of alloying elements such as Mn and trace microalloying additions. These differences may influence mechanical properties and corrosion behavior; however, the selected material provides a simplified and reproducible model system to investigate fundamental corrosion and MIC processes.

### 2.2 Experimental set-up

Prior to use, the seawater was autoclaved at 120 °C for 20 min, whereas the sediment sample was used as a natural microbial inoculum. This approach ensured controlled and reproducible initial conditions, with seawater primarily acting as the electrolyte phase and sediment providing the microbial consortia driving MIC processes. Prior to exposure, carbon steel coupons were cleaned by acid pickling in 2% HCl, followed by neutralization in 1.2 M NaOH. The coupons were then thoroughly rinsed with deionized water, rinsed with acetone to remove residual moisture, and dried under a stream of nitrogen gas (SP0775-2013, 2013). The cleaned coupons were then weighed using a precision analytical balance.

**Figure 1:**
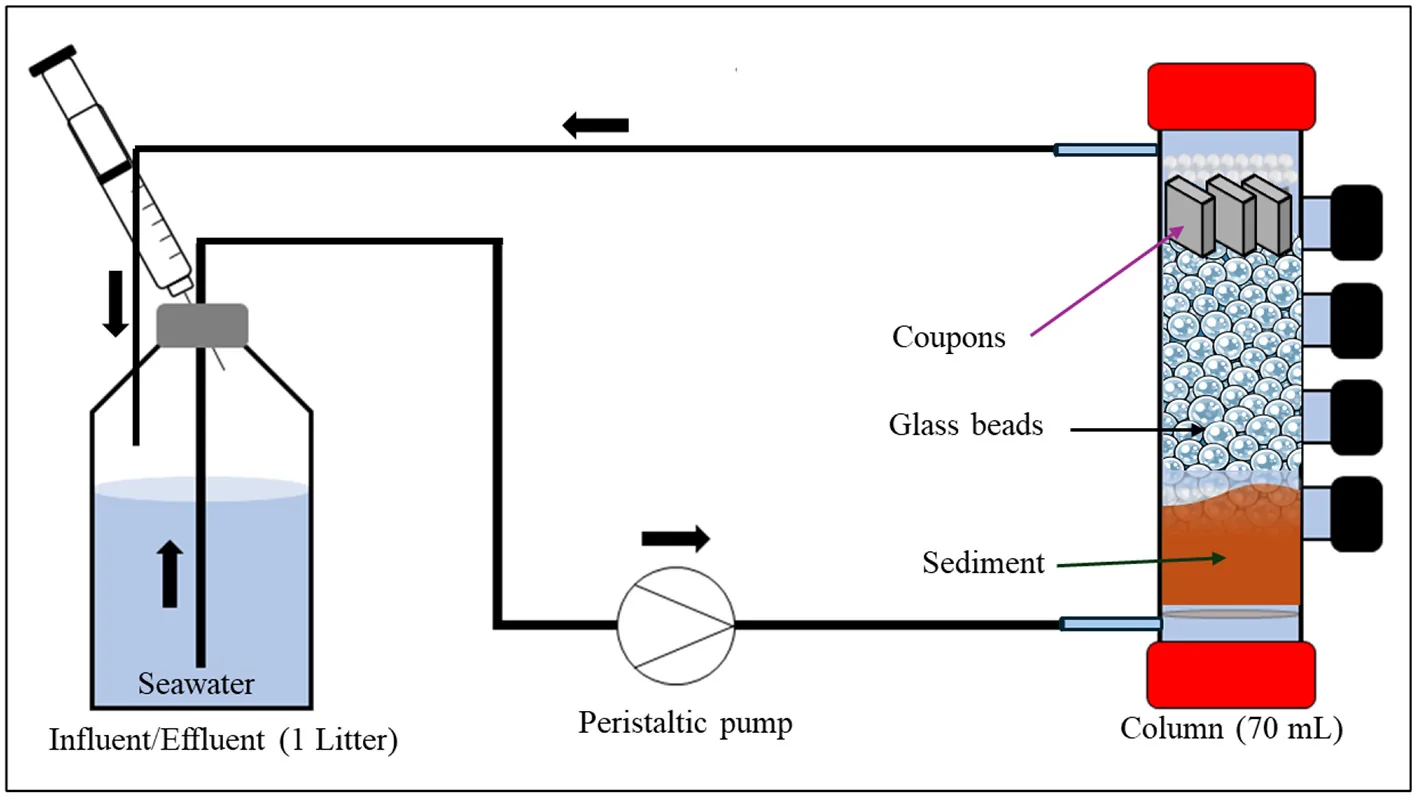
Schematic overview of the closed-loop multiport flow-column system; Three carbon steel coupons were suspended in the overlying water column. Coupons were separated using glass beads to prevent contact. The black lines indicate tubing. A syringe connected to the influent reservoir served as a pressure-relief outlet to accommodate potential gas production.

The experimental system (Figure 1) consisted of a closed-loop setup (using MINIPULS 3 Peristaltic Pumps, Gilson, USA) containing 5 mL of sediment as an inoculum, placed at the bottom of each column and 1 L of circulating sterilized seawater. Three nutrient treatments were investigated (yeast extract, sodium lactate, and no nutrient). In each column, three coupons were suspended in the overlying water column (Figure 1). Coupons were spatially separated using sterile glass beads (Ø = 0.238 cm) to prevent contact. Prior to assembly, columns and glass beads were autoclaved at 120 °C for 20 min, and all tubing was sterilized using 70% (v/v) ethanol. Anoxic conditions were established by purging the entire system with nitrogen gas prior to the start of the experiment. The setups were maintained under a fume hood at 21 ± 0.5 °C. The initial pH was 8.26. Initially, columns were inoculated and incubated under static conditions for 3 days to allow initial biofilm establishment, after which the system was operated at a flow rate of 0.05 mL min^−1^ and maintained for 25 days. The flow rate of 0.05 mL min^−1^ was selected to represent a low-flow, weakly mixed condition rather than turbulent bulk seawater flow. In offshore environments, hydrodynamic conditions near steel surfaces are highly heterogeneous. While exposed monopile surfaces may experience wave- and current-driven turbulent flow, regions near the seabed, within sediment-contact zones, and beneath biofilm or corrosion-product deposits are often governed by weaker shear and boundary-layer-controlled mass transport. Under such conditions, substrate delivery, metabolite retention, and biofilm development can differ substantially from well-mixed bulk flow environments, which is particularly relevant for MIC processes (Christiansen et al., 2026; Rivera-Alvarez et al., 2020).

Yeast extract (1 g L^−1^) and sodium lactate (3.5 g L^−1^) were added separately as organic carbon amendments. Each biotic treatment was prepared in triplicate, together with sterile abiotic controls. Sodium lactate was selected as a defined low-molecular-weight organic electron donor commonly used in sulfate-reducing bacteria (SRB) media and MIC studies, including formulations derived from Postgate media (Stein et al., 2025). Yeast extract was selected as a complex nutrient supplement frequently used to stimulate microbial growth and biofilm development in laboratory cultivation systems. While lactate represents a simple carbon source for microbial growth. The concentrations were chosen based on commonly applied ranges in MIC-related studies and were intended to compare the effects of simple vs. complex carbon amendments. Therefore, three treatments were considered: Blank/no nutrient, Lactate, and Yeast conditions.

### 2.3 Corrosion layer/biofilm analysis

After the experimental period, carbon steel coupons were carefully removed from the system for morphological and compositional analyses. To preserve the integrity of the biofilm and corrosion products, separate sets of coupons were used for biofilm/corrosion layer thickness measurements and for elemental analysis and focused ion beam–scanning electron microscopy (FIB–SEM), although all coupons originated from the same loop/bottle within each treatment. This approach minimized disturbance, oxidation, or alteration of the biofilm during repeated handling and preparation.

For biofilm and corrosion layer thickness analysis, coupons were gently washed once with filter-sterilized PBS/dH_2_O (1:2, v/v), then incubated overnight at 4 °C in 1 mL 2.5% (v/v) glutaraldehyde solution for fixation of microorganisms. Subsequently, the coupons were washed first with filter sterilized PBS/dH_2_O (1:2, v/v) for 5 min, then for another 5 min in sterilized dH_2_O. The dehydration of coupons was carried out using different ethanol dilutions (30% [30 min], 50% [30 min], 70% [30 min], 80% [60 min], 90% [60 min] (v/v) and absolute [60 min]) and dried with N_2_ gas (An et al., 2020). Surface morphology and corrosion layer thickness were then quantified using a 3D optical profilometer VR-3000 (Keyence Corporation, Osaka, Japan). Surfaces were analyzed by white-light interferometry and focus-variation techniques to generate high-resolution three-dimensional reconstructions. Surface and profile roughness parameters were extracted according to ISO 25178 (25178-6:2010, 2010), following form removal and Gaussian filtering (λc = 0.8 mm).

For elemental analysis, a second set of coupons was prepared using the same washing and dehydration protocol, followed by sputter-coating with a thin gold layer (~15 nm, Quorum Technologies, United Kingdom) to enhance conductivity. Energy-dispersive spectroscopy (EDS) was performed using a Phenom XL G3 Desktop SEM (Thermo Fisher Scientific Inc., Waltham, MA, USA) operated at 15 kV to determine the elemental composition of corrosion products and biofilm-associated material. The same coupons were used for FIB–SEM analysis. First, samples were examined by SEM to obtain surface images prior to sectioning. Cross-sections were sliced by FIB (FEI 200xP, Thermo Fisher Scientific Inc., Waltham, MA, USA) and imaged by SEM at an angle of 52° to the surface (5 kV, secondary electron detector)

### 2.4 Gravimetric analysis and surface profilometry

At the end of the experiment, the carbon steel coupons were cleaned following a standardized procedure (SP0775-2013, 2013). Coupons were first immersed in a 1:1 solution of DBT-HCl and deionized water for 10 min to remove corrosion products (vigorously shaking). They were then neutralized in a 1.2 M NaHCO_3_ solution for 5 min. Following neutralization, the coupons were thoroughly rinsed with deionized water, rinsed again with acetone to remove any remaining residues, and subsequently dried under a stream of nitrogen gas. Finally, the cleaned coupons were stored in a desiccator until further analysis.

#### 2.4.1 Weight loss measurements

The weight loss of the carbon steel coupons was determined to assess the extent of corrosion. In each experimental loop, a total of three carbon steel coupons were used. As it was not possible to reliably distinguish individual coupons in each loop after exposure due to the same shape and size, the combined weight of the three coupons was used for gravimetric analysis, representing one replicate per treatment. All coupons were included in the analysis, as the other methods applied were non-destructive. Prior to exposure, the initial weight of the three cleaned and dried coupons per column was recorded using an analytical balance (AW-224, Sartorius AG, Göttingen, Germany) with an accuracy of ±0.1 mg. After the experiment, the coupons were cleaned as explained above (removal of corrosion products and biofilm) and reweighed. The weight loss was calculated by subtracting the final weight from the initial weight. Corrosion rates were subsequently calculated based on the weight loss, the exposed surface area of the coupons, the duration of exposure, and the material density, according to the Equation 1:


$$\begin{array}{l} Corrosion\;Rate\;\left(\frac{mm}{year}\right)=\frac{87.6\;\times \;\Delta W}{\rho \;\times \;A\;\times \;t} \end{array}$$ (1)

Where, ΔW is the weight loss (mg), ρ is the density of carbon steel (g/cm^3^), A is the surface area of the coupon (cm^2^), and t is the exposure time (hours) (An et al., 2020). Statistical analysis was performed using a non-parametric Kruskal–Wallis test due to the small sample size and non-normal distribution of the data. *Post-hoc* comparisons were conducted using Dunn's test with Bonferroni correction.

#### 2.4.2 3D profiling analysis

After cleaning, selected carbon steel coupons were analyzed using a NanoFocus μSurf expert (Keyence, Germany) to characterize surface topography and quantify pitting. Surface scans were performed in non-contact mode at a lateral resolution of 0.64 μm and a vertical resolution of up to 10 nm. Representative areas of each coupon were scanned to capture the distribution of pits and surface roughness. Analysis was conducted using the μSurf Analysis premium software (v.10), extracting parameters such as maximum pit depth (mm), pit density, and surface roughness values (Sa, Sq) according to ISO 25178 standards (25178-6:2010, 2010). For each coupon, the entire surface topography was captured in a single scan. The outer edges of the samples were excluded from subsequent analysis to facilitate data evaluation, and all measurements were performed under identical scanning conditions.

### 2.5 Metabolomics analysis

Metabolomic profiling was conducted on samples collected from the circulating water of the experimental systems to investigate metabolic changes associated with microbial activity and corrosion processes. At the end of the experiment, water samples were collected (from all replicates) and immediately mixed with methanol at a ratio of 0.5 mL sample to 1 mL methanol (Kotu et al., 2024). After thorough mixing, the extracts were kept at −20 °C until subsequent analysis. Metabolite analysis was performed using liquid chromatography–mass spectrometry (LC–MS) by connecting an Agilent 1290 Infinity II (Agilent Technologies, Waldbronn, Germany) to an electrospray ionization (ESI) source with a TripleTOF 6,600 high resolution mass spectrometer (Sciex, Darmstadt, Germany). Instrument calibration was performed after every 5th sample to ensure mass accuracy < 2 ppm. The recorded mass range was 80–1,200 Da in positive and negative ion mode, using a source temperature of 300 °C and recording MS/MS scans in information-dependent acquisition (IDA) mode. For chromatographic separation, 10 μL were injected into an Agilent Zorbax Eclipse XDB C18 column (2.1 mm × 100 mm, particle size 1.8 μm) heated to 40 °C. The mobile phase A consisted of 0.1% FA aqueous solution and B consisted of ACN (also 0.1% FA), starting with 90% A held for 1 min, increased to 99% B within 7 min and held for additional 3.9 min. This was followed by a decrease back to 90% A within 0.1 min and held for 3 min. The full analysis time was 15 min. The flow rate was set at 0.5 mL/min. Data processing, including peak detection, alignment, and normalization, was carried out using MS-Dial software (ver.4.9.221218) (Tsugawa et al., 2015). Metabolite identification was achieved by comparison against version 17 of the ExpBioInsilico MS Dial public libraries (https://systemsomicslab.github.io/compms/msdial/main.html), containing negative and positive spectra respectively. This would be equivalent to levels 2 and 3 annotation confidence for analytes with and without recorded MS/MS spectra respectively, following the schematic of Schymanski et al. (2014). Statistical analyses were performed to identify significant differences in metabolite profiles between treatments. Mean peak intensities of replicates for each nutrient were calculated and expressed relative to the values obtained in lactate samples. To ensure that reported compounds are of biological origin, only compound peaks with intensities at least 100-fold higher compared to control samples (sterile) were retained in the analysis.

### 2.6 Dissolved sulfide measurement

Total dissolved sulfide in the aqueous phase was quantified after 28 days using the methylene blue method and is reported as ΣH_2_S (H_2_S + HS^−^) in mM, expressed as H_2_S equivalents, because the assay is performed under acidic conditions that convert HS^−^ to H_2_S (Nagy et al., 2014). Sulfide was used as a chemical indicator of sulfidogenic activity in the cultures. For each treatment (biotic and abiotic), three replicates were analyzed, each representing an independent experimental loop. One sample was collected per replicate, taken from the bulk influent/effluent reservoir in the flow systems. Statistical analyses were performed in Python (Google Colab). Prior to one-way ANOVA, the data were screened for major departures from normality (Shapiro–Wilk test) and homogeneity of variances (Levene's test). As no evidence for violation of these assumptions was detected (*p* > 0.05), a one-way ANOVA was used to evaluate differences in ΣH_2_S among treatments. Where appropriate, Tukey's HSD test was used for *post-hoc* pairwise comparisons. Final pH was measured immediately after disconnecting each system to capture end-point chemical conditions.

### 2.7 16S rRNA sequence analysis

#### 2.7.1 DNA extraction

Part of initial sediment (5 mL) was collected in a sterile falcon tube at the start of the experiment and immediately stored at −80 °C to enable direct comparison with post-exposure samples. At the end of the experiment (28 days), each system was disassembled under sterile conditions, and biofilm samples were collected from coupons in the biotic treatments. For each treatment, one coupon per replicate (loop) was used for swabbing. No biofilm sampling was performed on abiotic coupons. The persistence of abiotic conditions was indirectly assessed by monitoring sulfide production and metabolite profiles; the absence of detectable H_2_S and metabolites throughout the experiment was used as an indicator of no microbial activity. Following biofilm collection, swabs were placed in sterile tubes and supplemented with DNA Shield to preserve nucleic acid integrity during storage and transport. Then they were immediately frozen at −80 °C. Preserved samples were then shipped to the sequencing provider (Greengate Genomics GmbH, Potsdam, Germany) in Dry ice for downstream DNA extraction and analysis. Total DNA from swab samples was extracted using the PowerSoil Pro kit (Qiagen, Hilden, Germany) following the manufacturer's protocol. DNA concentrations were quantified using a Qubit™ fluorometer with the Qubit™ 1X dsDNA BR Assay Kit (Invitrogen, Thermo Fisher Scientific Inc., Waltham, MA, USA).

#### 2.7.2 Amplification and sequencing of 16S rRNA gene

For each DNA extraction sample, two technical replicates were sequenced. The amplification of the 16S rRNA gene sequences was performed using universal primers for bacteria 515F (5′ GTGTGYCAGCMGCCGCGGTAA3′) and 806R (5′ CCGGACTACNVGGGTWTCTAAT3′) with specific barcodes attached to the primers. Prior to sequencing, all PCR products were purified using a magnetic beads Agencourt^®^ AMPure^®^ XP- Kit (Beckman Coulter Life Sciences, Krefeld, Germany) according to the protocol. Purified PCR amplicons from all samples were pooled in equimolar ratios. The pool was sent for Illumina paired-end sequencing at Novogene (Novogene GmbH, Munich, Germany) on a NovaSeq6000 machine with 250 PE protocol (2 × 250 paired-end reads).

#### 2.7.3 Bioinformatic analysis

The sequencing library was demultiplexed using cutadapt v3.4 (Martin, 2011) using the following parameters: –e 0.2 –q 15,15 –m 150—discard-untrimmed. The ASVs were generated using trimmed reads and the DADA2 package v1.20 (Callahan et al., 2016) with R v4.1 using the pseudo-pooled approach with the following non-default parameters: maxN = 10, minLen = 80. Final ASVs were obtained by read merging and chimera removal using DADA2 functions. Taxonomic assignment was performed using DADA2 and the SILVA database v138.1 (Quast et al., 2012). Subsequently, ASVs representing chloroplasts, mitochondria and singletons were removed. Samples were subsampled to 20,000 reads using the vegan package v2.6-4 (https://cran.r-project.org/package=vegan). Statistical analysis and visualization were performed in R v4.1 using the following packages: phyloseq v1.38.0 (McMurdie and Holmes, 2013), ggplot2 v3.5.2 and vegan v2.6-4 (Wickham, 2016).

## 3 Results

### 3.1 Corrosion layer/biofilm thickness

**Figure 2:**
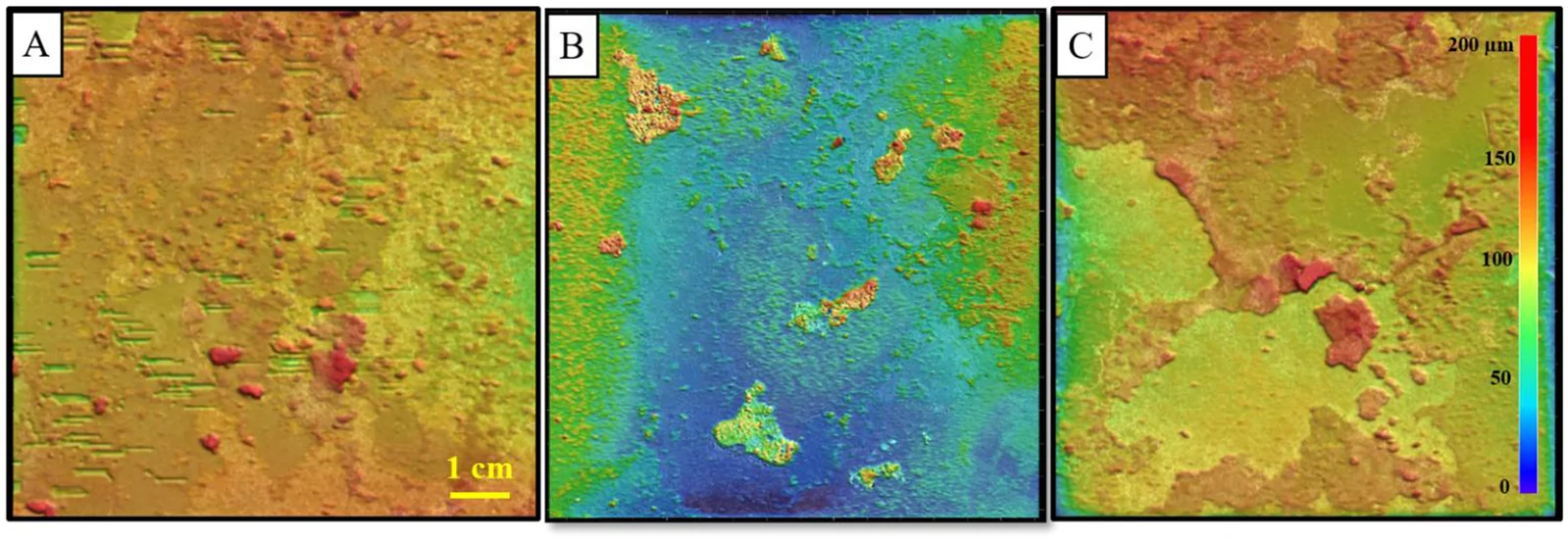
Thickness distribution of the corrosion product layer/biofilm on carbon steel coupons exposed to different nutrient treatments (resolution: 0.02 mm). **(A)** Blank. **(B)** Lactate. **(C)** Yeast.

**Figure 3:**
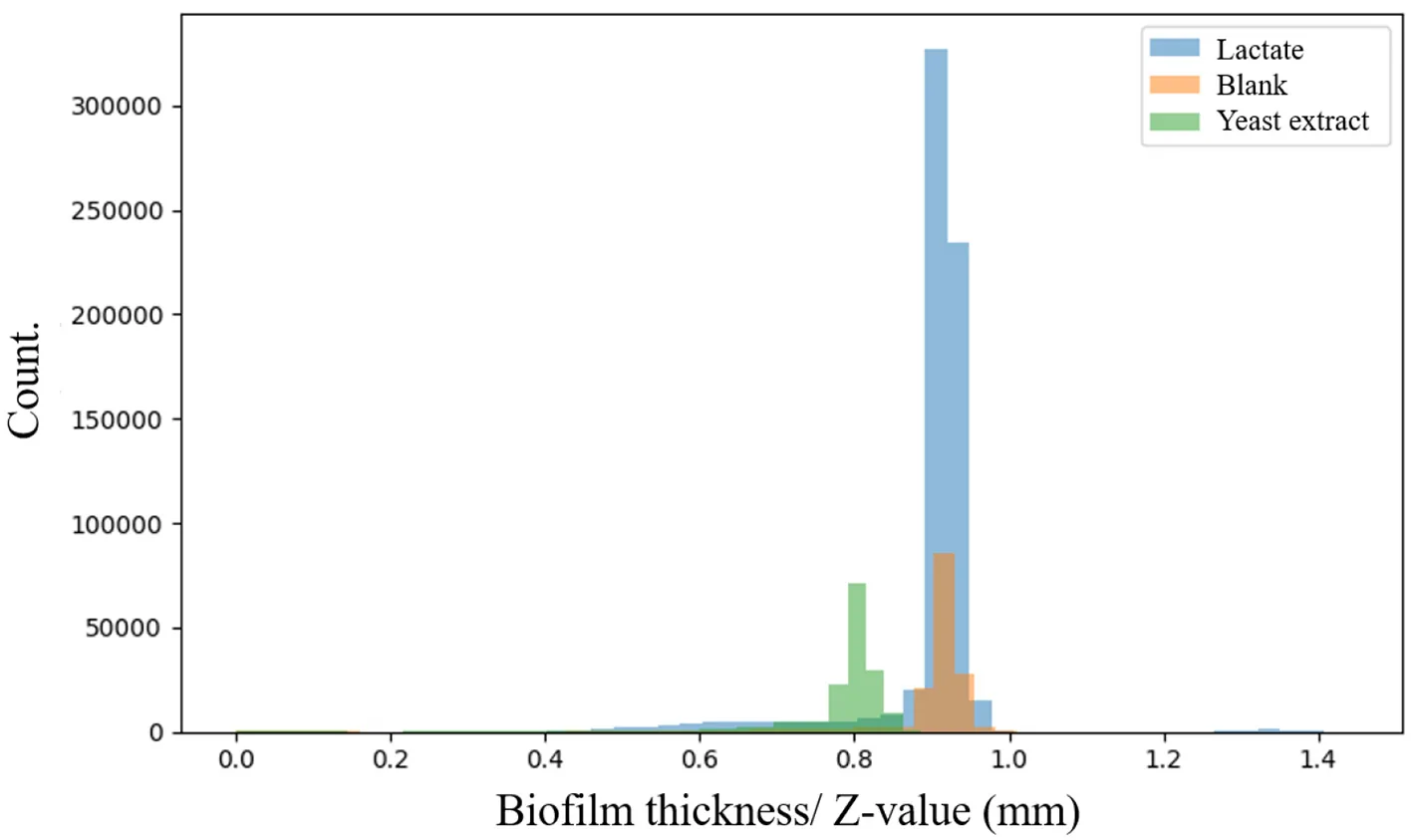
Distribution of corrosion layer/biofilm thickness values measured by 3D optical profilometry. Frequency histograms of extracted Z-values are shown for carbon steel coupons exposed to lactate, blank/no-nutrient, and yeast extract conditions. The reported Z-values, representing biofilm thickness, were calculated based on the combined biofilm thickness measured on both sides of each coupon.

Corrosion layer/biofilm thickness on exposed carbon steel coupons was evaluated using 3D optical profilometry (Figure 2). In the representative analyzed coupons, Lactate showed the highest mean Z-value (0.897 μm) followed by the blank/no-nutrient condition (0.878 μm) and yeast extract (0.761 μm). Median values were similar for lactate and blank (0.914 and 0.915 μm, respectively), but lower for yeast extract (0.797 μm). The Z-value frequency distribution (Figure 3) showed a narrow and intense peak for lactate at higher thickness values, approximately 0.90–0.95 μm, indicating a relatively uniform and coherent surface layer. The blank treatment also peaked in a similar range but showed a broader distribution, suggesting greater heterogeneity. Yeast extract showed its main peak at lower Z-values, approximately 0.78–0.82 μm, consistent with a thinner surface layer.

Variability metrics supported these observations (Table 1). Lactate showed the lowest coefficient of variation (0.098) compared with 0.157 for blank and 0.172 for yeast extract, indicating the most coherent surface coverage. Yeast extract showed the lowest thickness and highest relative variability, suggesting a thinner and more heterogeneous corrosion layer/biofilm. Although statistical comparison of the Z-value distributions indicated significant differences among treatments, the analysis is based on one technical replicate per treatment. Therefore, these results should be interpreted as descriptive evidence of treatment-associated differences in the measured coupons, and further biological replicates are required to confirm treatment-level significance.

**Table 1:** Descriptive statistics of corrosion layer/biofilm thickness measured by 3D optical profilometry.

Treatment	Count	Mean (mm)	Median (mm)	SD	max	IQR	CV	Interpretation
Lactate	667,764	0,897	0,914	0,087	1,436	0,022	0,098	Thickest mean and most coherent
Blank (no nutrient)	160,545	0,878	0,915	0,138	1,328	0,025	0,157	Similar median to lactate, but more heterogeneous
Yeast extract	166,721	0,761	0,797	0,131	1,198	0,029	0,172	Thinnest and most heterogeneous

Extracted Z-values from both side of carbon steel coupon surfaces were used to compare layer thickness among lactate, blank/no-nutrient, and yeast extract treatments. Mean and median values describe the central thickness, while standard deviation (SD), interquartile range (IQR), and coefficient of variation (CV) indicate spatial variability and coherence of the surface layer.

### 3.2 Elemental analysis of the corrosion products

**Figure 4:**
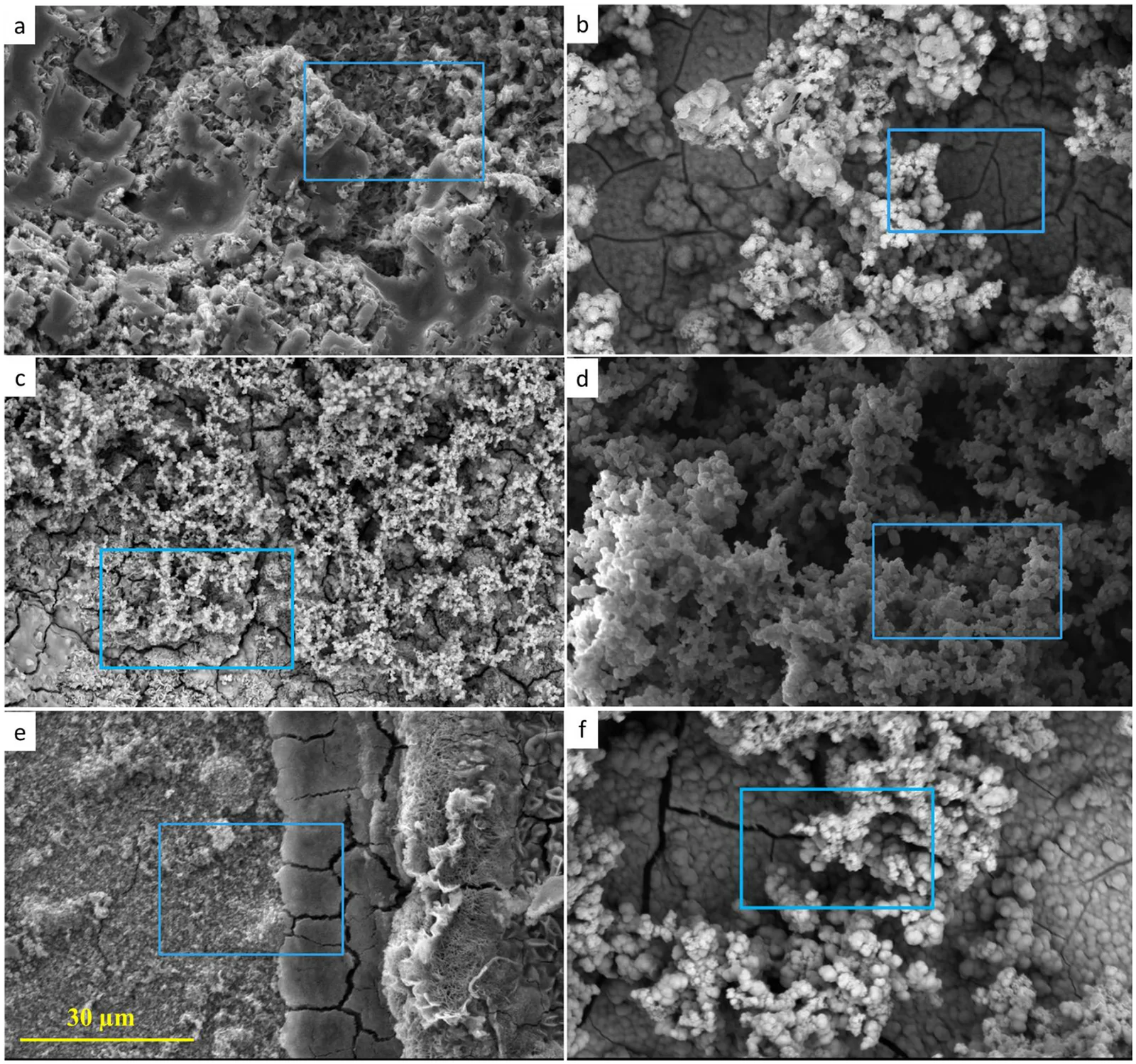
SEM images of the areas used for the corresponding EDS analyses. **(a)** Blank-Biotic. **(b)** Blank-Abiotic. **(c)** Lactate-Biotic. **(d)** Lactate-Abiotic. **(e)** Yeast-Biotic. **(f)** Yeast-Abiotic.

Representative EDS spectra of the corrosion product layer formed on carbon steel coupons after 28 days of immersion under different treatment conditions are presented in Table 2. Figure 4 shows SEM images of the areas used for the corresponding EDS analyses presented in Table 2. In the evaluated coupons, the relative sulfur concentration within the biofilm/corrosion layer was found to be higher in coupons exposed to the yeast condition compared to the other treatments (two to five times higher). In contrast, the lowest sulfur contents were detected in the lactate. While EDS cannot be used to identify specific Fe–S mineral phases, nor to distinguish among pyrite, greigite, pyrrhotite, or FeS, the observed Fe/S trends provide meaningful qualitative evidence regarding the relative abundance of sulfide-rich vs. sulfide-poor corrosion products (Duan and Sun, 2023). The Fe/S ratio indicated an inverse relationship with the sulfur concentration across treatments, with higher sulfur levels corresponding to lower Fe/S ratios. A lower Fe/S ratio in corrosion products indicates a higher proportion of sulfur relative to iron, which suggests the formation of sulfur-rich iron sulfide phases such as pyrite (FeS_2_), greigite (Fe_3_S_4_), or iron-deficient pyrrhotite (Fe_1_-XS) rather than iron-monosulfide (FeS). Overall, the EDS analysis indicates that nutrient availability influenced the elemental composition of the corrosion layers, particularly with respect to sulfur accumulation. However, definitive characterization of corrosion product mineralogy would require phase-specific analytical techniques such as Raman spectroscopy or X-ray diffraction (XRD). In contrast, corrosion products formed on abiotic control coupons showed a lower sulfur content and much higher Fe/S ratio.

**Table 2:** Representative energy-dispersive X-ray spectroscopy (EDS) results of corrosion product layer formed on carbon steel coupons after 28 days of exposure under different nutrient conditions.

Treatments	Element (Weight Conc.)						
	Carbon	Oxygen	Sodium	Phosphorus	Sulfur	Iron	Fe/S
Blank-Biotic	3.2	19	6.6	1.3	10	57.8	5.7
Lactate-Biotic	2.3	22.9	5.3	16.7	4.5	48.3	10.71
Yeast-Biotic	4.2	16.1	8.1	0.9	21.7	49	2.3
Blank-Abiotic	1.3	45	1.5	0.8	0.4	50	125
Lactate-Abiotic	0.9	38	7.2	8.4	0.5	43	86
Yeast-Abiotic	2.3	45	4.4	8.3	0.3	39	130

### 3.3 Cross-sectional analysis of corrosion product/biofilm layer

**Figure 5:**
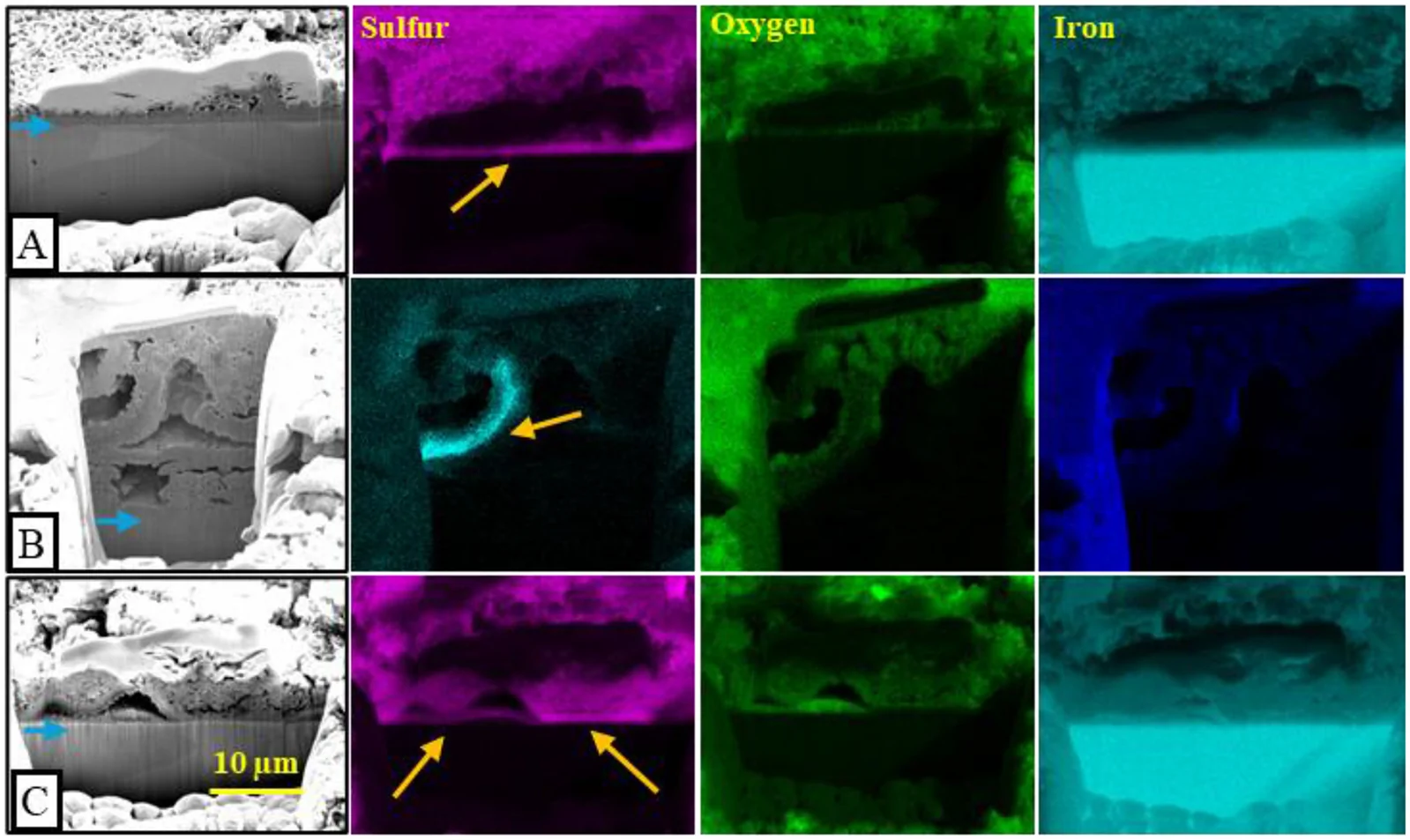
Focused ion beam–scanning electron microscopy (FIB–SEM) cross-sectional analysis of corrosion products formed on carbon steel under different nutrient treatments. **(A)** Blank (no nutrient), **(B)** lactate, and **(C)** yeast extract. Orange arrows indicate sulfur-enriched regions. Blue arrows indicate the line separating the corrosion product and the metal surface.

Cross-sections of the corrosion products and biofilm layers were prepared using FIB-SEM (Figure 5). Each section represents a small and localized surface area. Nevertheless, comparable structural features were repeatedly observed across different samples. These recurring patterns may provide insight into processes associated with MIC. In the analyzed specimens, particularly in the lactate and yeast treatments, the deposits appeared porous and layered rather than dense and compact (Figure 5). The layers contained interconnected voids and channel-like pathways extending from the steel surface toward the bulk medium. Similar porous and channelized corrosion deposits have been reported in previous studies and associated with localized anodic activity combined with limited mass transport (Mielke et al., 2011). The internal porosity likely allows inward transport of aggressive species, such as chloride ions and residual oxygen, while enabling outward diffusion of Fe^2+^ ions. This exchange can sustain the localized electrochemical reactions at the interface (Wang et al., 2026). The heterogeneous and channelized nature of the deposits is consistent with under-deposit corrosion and uneven metal dissolution typical of pitting or crevice-type attack beneath biofilms or sediments (Yang et al., 2024; Meng et al., 2008). However, these patterns may also reflect processes related to the transport of nutrients and metabolic waste products within the deposit structure.

EDS mapping of the cross-sections (Figure 5, orange arrows) suggested a heterogeneous sulfur distribution within the corrosion layers, with localized sulfur enrichment observed inside cavities and interconnected pores, particularly near the metal surface. In these regions, lower oxygen signals were also detected. Although EDS does not provide phase-specific information, the observed sulfur–oxygen distribution patterns suggest chemically heterogeneous microenvironments within the biofilm–corrosion matrix. Similar sulfur-enriched regions have previously been reported in biofilm-associated corrosion systems and are commonly associated with localized corrosion processes involving sulfur-containing deposits (Enning and Garrelfs, 2014; Enning et al., 2012).

### 3.4 Weight loss measurements and 3D profiling

**Figure 6:**
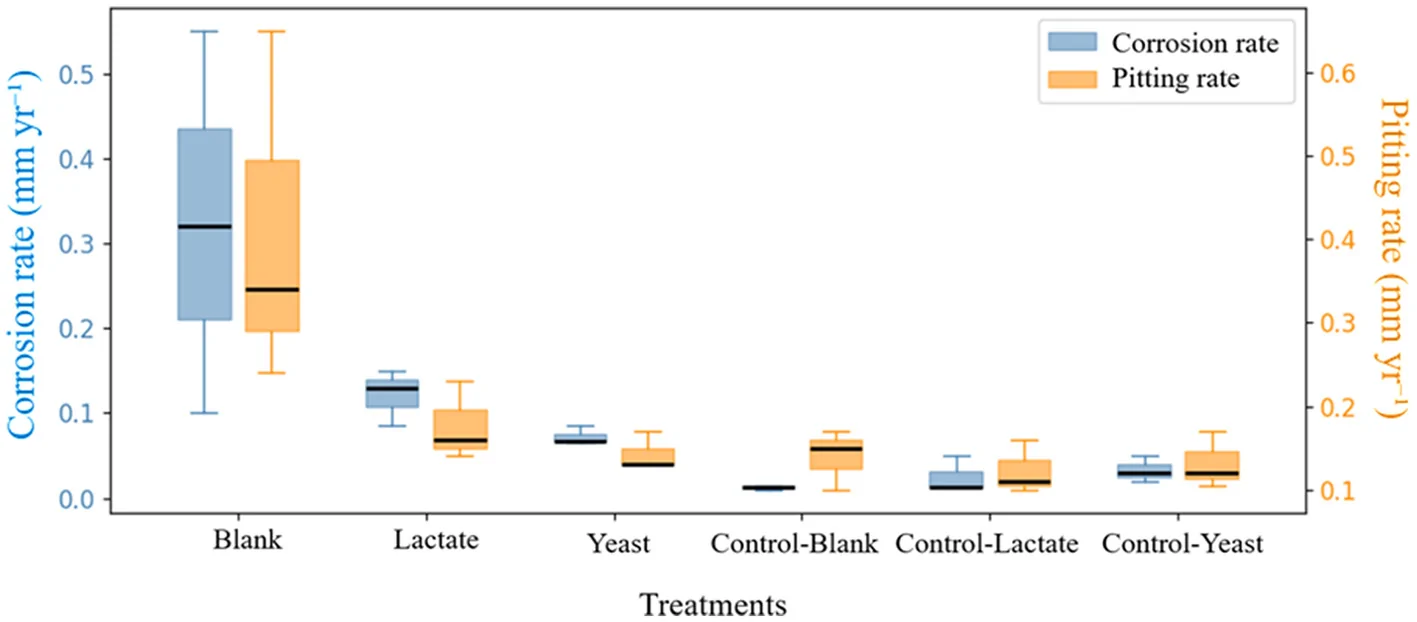
Corrosion rate (blue, left y-axis) and pitting rate (orange, right y-axis) measured on carbon steel coupons exposed under different nutrient treatments (Blank (no nutrient added), Lactate, and Yeast extract). The sterile controls did not show any clear difference among different nutrient conditions. Corrosion rates in all sterile treatments remained low, ranging approximately from 0.01 to 0.05 mm yr^−1^.

The corrosion rates of carbon steel coupons exposed to the different nutrient treatments are presented in Figure 6. The highest corrosion rates were observed under the biotic blank/no-nutrient condition, with a mean value of 0.323 ± 0.225 mm yr^−1^ and a median of 0.320 mm yr^−1^. This treatment also showed the largest variability among replicates, with individual corrosion rates reaching 0.55 mm yr^−1^. In contrast, coupons exposed to lactate and yeast extract showed lower corrosion rates, with mean values of 0.122 ± 0.033 and 0.072 ± 0.011 mm yr^−1^, respectively. Thus, compared with the blank condition, the mean corrosion rate was approximately 2.7-fold lower in the lactate treatment and 4.5-fold lower in the yeast extract treatment. The Kruskal–Wallis test showed a potential strong correlation in corrosion rates, although the overall difference did not reach the conventional significance threshold (*H* = 5.535, *p* = 0.0628). Pairwise Mann–Whitney U tests with Bonferroni correction did not show significant differences between individual treatment pairs.

Pitting corrosion was evaluated using 3D optical profilometry of the exposed carbon steel coupon surfaces. Surface reconstructions were processed using μSurf Analysis Premium software (v.10) to obtain quantitative pit parameters, including maximum pit depth, pit area, and pit volume. Pitting rates were calculated from the deepest pit measured for each coupon and are shown in Figure 6. The highest pitting rates were again observed under the biotic blank/no-nutrient condition, with a mean value of 0.410 ± 0.214 mm yr^−1^ and values ranging from 0.24 to 0.65 mm yr^−1^. Lactate- and yeast-extract-treated samples exhibited lower pitting rates, with mean values of 0.177 ± 0.047 and 0.143 ± 0.023 mm yr^−1^, respectively. Compared with the blank treatment, the mean pitting rate was approximately 2.3-fold lower in the lactate treatment and 2.9-fold lower in the yeast extract treatment. Statistical analysis supports an overall treatment effect on pitting rate, as determined by the Kruskal–Wallis test (*H* = 6.006, *p* = 0.0496). Pairwise Mann–Whitney U tests with Bonferroni correction did not identify significant differences between individual treatment pairs, indicating that the overall effect reflects the combined treatment-level distribution rather than a single dominant pairwise contrast. Together, the corrosion and pitting results show that the blank/no-nutrient condition produced the highest degradation response, whereas lactate and yeast extract were associated with consistently lower corrosion and pitting rates. This pattern is consistent with enhanced corrosion under carbon-limited conditions, as reported previously (Cui et al., 2025; Li et al., 2021; Pu et al., 2023; Yuxuan et al., 2022). The sterile controls did not show any clear difference among different nutrient conditions. Corrosion rates in all sterile treatments remained low, ranging approximately from 0.01 to 0.05 mm yr^−1^. This indicates that, in the absence of microorganisms, the different nutrient amendments had only a limited effect on carbon steel corrosion under the tested conditions.

**Figure 7:**
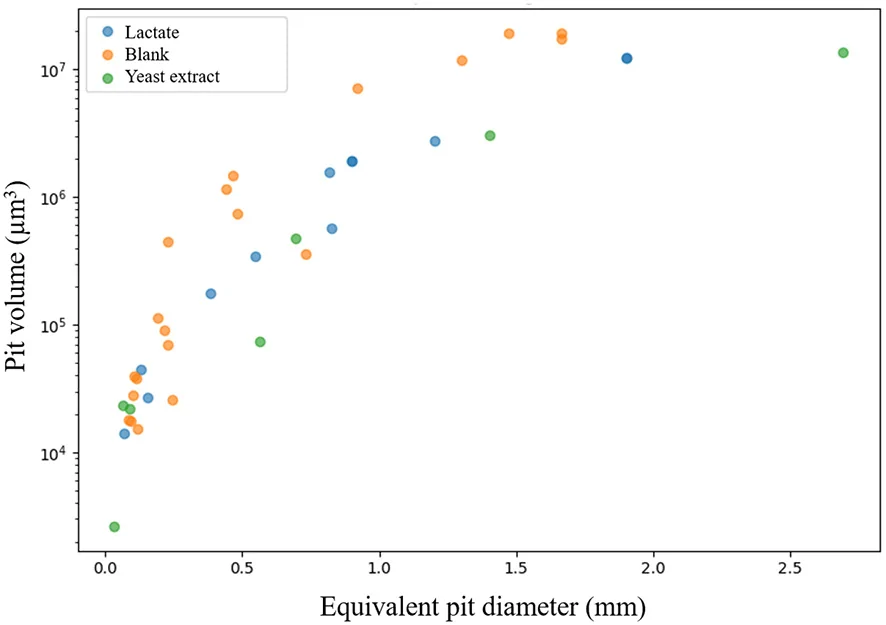
Relationship between equivalent pit diameter and pit volume for deep pits (≥ 10 μm) identified by 3D optical profilometry. Each point represents an individual pit detected on carbon steel coupons exposed to lactate, blank/no-nutrient, or yeast extract conditions, based on three technical replicates per treatment. Pit volume is shown on a logarithmic scale to visualize the wide range of pit sizes.

Pit morphology was further evaluated by plotting pit equivalent diameter against pit volume for pits with a maximum depth greater than 10 μm (Figure 7). Across all treatments, pit volume increased with increasing equivalent diameter, indicating that larger pit openings were generally associated with greater material loss. The use of a logarithmic scale for volume highlights the broad range of pit sizes detected, spanning several orders of magnitude. The blank/no-nutrient treatment contained several pits with relatively large equivalent diameters and high volumes, including multiple points in the upper range of the distribution. This indicates that carbon-starved conditions were associated with the formation of large-volume pits. Lactate-treated samples also contained some deep pits with high volumes, although most lactate-associated pits were distributed across intermediate equivalent diameters and volumes. Yeast extract showed fewer pits overall, but included isolated large pits with high volume, indicating that while the number of selected deep pits was lower, localized severe pitting still occurred. Overall, the distribution suggests that the blank/no-nutrient condition produced a higher occurrence of large-volume pits, whereas lactate and yeast extract showed fewer high-volume features. These data support the pitting-rate results, where the blank condition showed the highest pitting response. However, the presence of isolated large pits in all treatments indicates that localized corrosion was not completely suppressed by nutrient addition.

### 3.5 Metabolomics

Untargeted metabolomic profiling suggested clear differences in metabolite abundance among nutrient treatments (Supplementary Figure S1). Although metabolite annotation confidence in untargeted metabolomics remains limited, several compounds were consistently enriched in the yeast treatment relative to the lactate and blank conditions. These differences likely reflect broader microbial metabolic activity associated with the availability of complex organic substrates. Among the detected compounds, purine, acetosyringone, fusaric acid, 2-hydroxyacetophenone, levodopa, betaine, and isovaleric acid showed increased relative intensities in the yeast treatment. Many of these metabolites are commonly associated with microbial processing of complex organic matter, amino acid metabolism, osmotic regulation, or fermentation-related pathways (Grove, 2025; Kennelly et al., 2024; Subramoni et al., 2014; Engl et al., 2026; Tung et al., 2017; Bavi et al., 2022; Liu et al., 2026; Salazar et al., 2022). In contrast, the blank and lactate treatments showed fewer distinctive metabolites, likely reflecting lower metabolic complexity and more limited substrate diversity. However, because yeast extract itself represents a chemically complex substrate, some detected metabolites may also originate from the medium background or from non-corrosion-related microbial activity. Guan et al. (2024) demonstrated that removing yeast extract from SRB culture media increased the corrosion rate of steel by approximately four-fold, which is in agreement with the observations of the present study. These findings suggest that complex nutrient amendments can substantially influence microbial metabolic profiles and should therefore be interpreted cautiously when assessing corrosion-related mechanisms.

### 3.6 Effect of flow on H_2_S production

**Figure 8:**
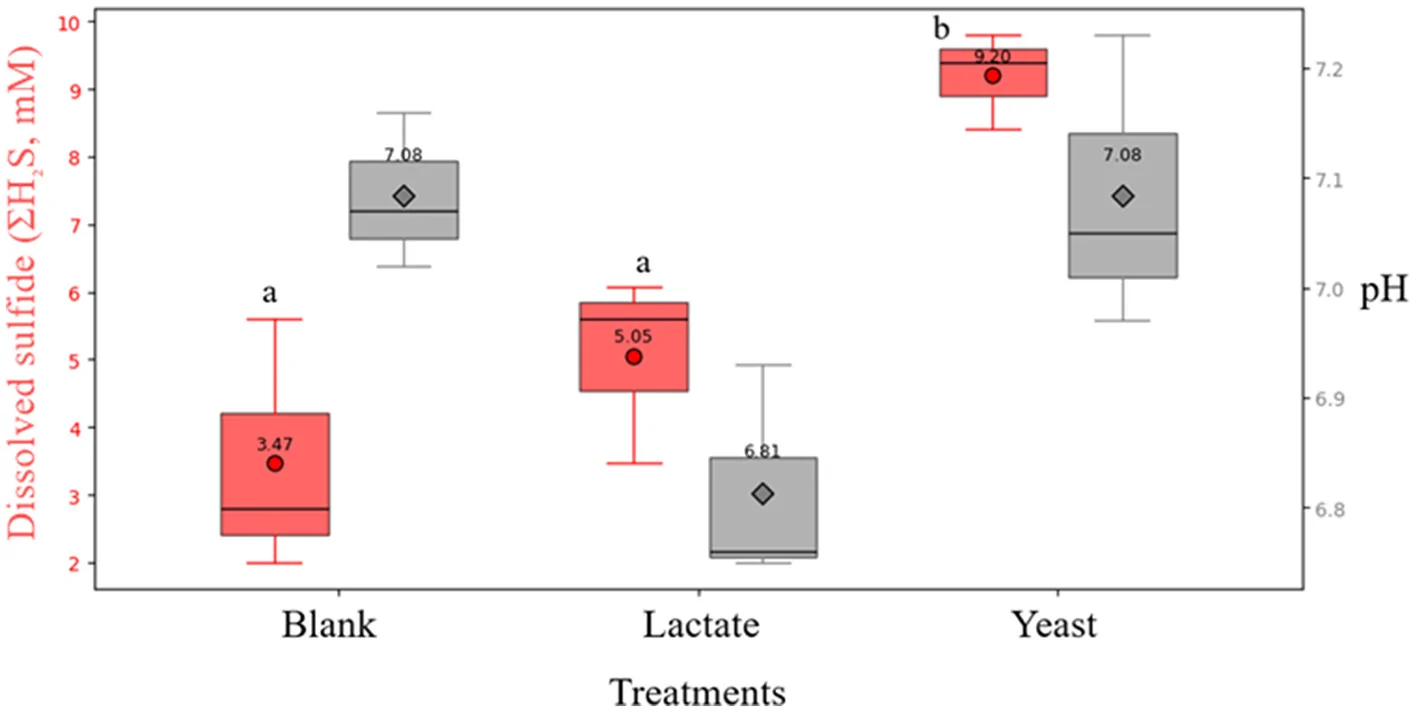
Dissolved sulfide concentrations under different nutrient treatments. Boxes show the interquartile range with median (line) and individual replicates (points). Different letters denote significant differences in dissolved sulfide concentrations between treatments (Tukey HSD, *p* < 0.05). pH change after 28 days of exposure (initial pH: 8.26).

The bulk medium acidified in all treatments over the course of incubation (Figure 8). Final pH values were 7.08 in the blank/no-nutrient condition, 6.81 in the lactate treatment, and 7.08 in the yeast extract treatment. Because sulfide speciation is pH-dependent, the relative distribution between HS− and molecular H_2_S was estimated from the final pH using the first dissociation equilibrium of H_2_S (Reese et al., 2011). Based on this calculation, the estimated H_2_S fraction was approximately 45% in the blank/no-nutrient and yeast extract treatments, and approximately 61% in the lactate treatment. Total sulfide concentrations, reported as ΣH_2_S, differed significantly among treatments, indicating that nutrient regime strongly influenced sulfate-reducing activity (Kruskal–Wallis, χ^2^ = 13.54, *p* = 0.0011). The yeast extract treatment reached the highest sulfide concentrations, with median values exceeding 9 mM. *Post-hoc* Dunn's test suggested that sulfide concentrations in the yeast extract treatment were significantly higher than those in both the blank/no-nutrient and lactate treatments (*p* < 0.05), while no significant difference was detected between blank/no-nutrient and lactate treatments (*p* = 0.18).

Importantly, total sulfide concentration, estimated H_2_S fraction, and corrosion severity did not follow the same treatment pattern. Yeast extract produced the highest total sulfide accumulation, but not the highest corrosion or pitting rates. Lactate had the highest estimated H_2_S fraction due to its lower pH, but corrosion and pitting rates remained lower than those observed under the blank/no-nutrient condition. In contrast, the blank/no-nutrient treatment showed the highest corrosion and pitting response despite having lower total sulfide than yeast extract and a lower estimated H_2_S fraction than lactate. This mismatch suggests that neither total sulfide concentration nor the estimated molecular H_2_S fraction alone explained the observed corrosion severity. However, dissolved sulfide represents only an end-point bulk aqueous measurement and does not capture the sulfur chemistry within the biofilm/corrosion-product microenvironment. Other sulfur species, including sulfate, sulfite, thiosulfate, polysulfides, and localized sulfide gradients, were not quantified and may also have influenced the observed corrosion response.

### 3.7 Characterization of microbial community of biofilms by 16S rRNA gene amplicon sequencing

Microbial community composition was analyzed using 16S rRNA gene amplicon sequencing, which targets conserved regions of the 16S rRNA gene, a widely used phylogenetic marker for prokaryotes. After sequencing, reads were denoized and grouped into amplicon sequence variants (ASVs). ASVs represent unique DNA sequences obtained after correcting sequencing errors and provide high-resolution information about microbial taxa, often close to species level. However, an exact match to species is not always possible because different species may share the same 16S rRNA sequence, and a single organism can contain multiple slightly different copies of this gene. Alpha diversity analysis showed comparable microbial richness and diversity across the three nutrient treatments (Supplementary Figure S2). The Observed and Chao1 richness indices exhibited overlapping distributions among the blank, lactate, and yeast treatments, indicating similar levels of species richness. Likewise, the Shannon diversity index showed only minor variation between treatments, although the yeast treatment tended to display slightly higher Shannon values.

**Figure 9:**
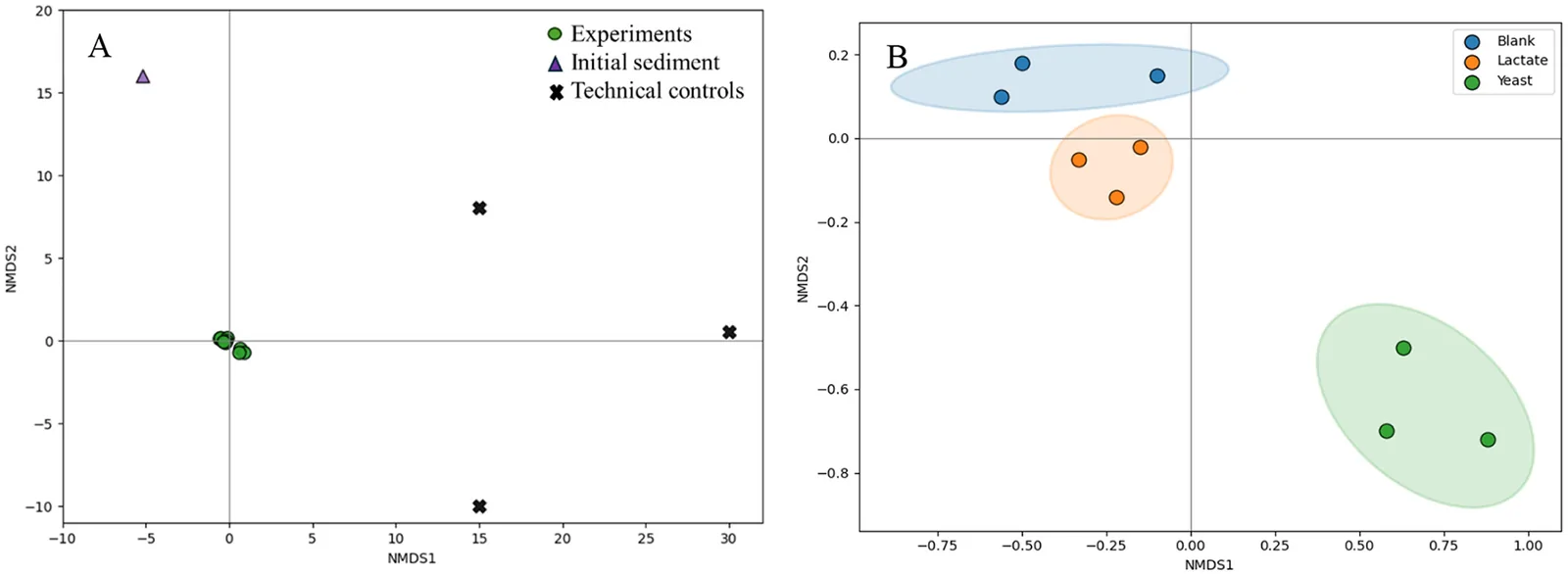
Beta diversity shown in NMDS plots. **(A)** Technical controls and the initial sediment used as an inoculum are very different from the rest of the samples. **(B)** Beta diversity shown in NMDS plots without controls and initial sediment (95% confidence ellipses).

Beta-diversity was evaluated using Bray–Curtis dissimilarity and visualized with non-metric multidimensional scaling (NMDS). The ordination showed a clear separation between the initial sediment inoculum and all incubated samples (Figure 9A), indicating that the microbial community changed substantially during the incubation period. To better compare the experimental treatments, a second NMDS analysis was performed after removing the initial inoculum and abiotic controls (Figure 9B). This plot showed a clear grouping of samples according to nutrient treatment. Communities from the lactate and blank treatments clustered closer together, while yeast-treated samples formed a separate group, suggesting that yeast created a stronger selective pressure on microbial community composition than lactate. Microbial community composition differed among nutrient treatments, as assessed by Bray–Curtis PERMANOVA using the Low-Water ASV abundance profiles. Treatment explained a large proportion of the variation in community composition (PERMANOVA, *R*^2^ = 0.846, *F* = 16.42, *p* = 0.0028). Homogeneity of multivariate dispersion was assessed using PERMDISP and was not significant (*p* = 0.1965), indicating that the PERMANOVA result was not primarily driven by unequal within-treatment dispersion. Pairwise PERMANOVA showed strong separation between treatment groups, although pairwise *p*-values did not remain significant because only three replicates were available per treatment, limiting the number of possible exact permutations. To identify which taxa contributed to these differences, relative abundances were analyzed using stacked bar plots at the genus level.

**Figure 10:**
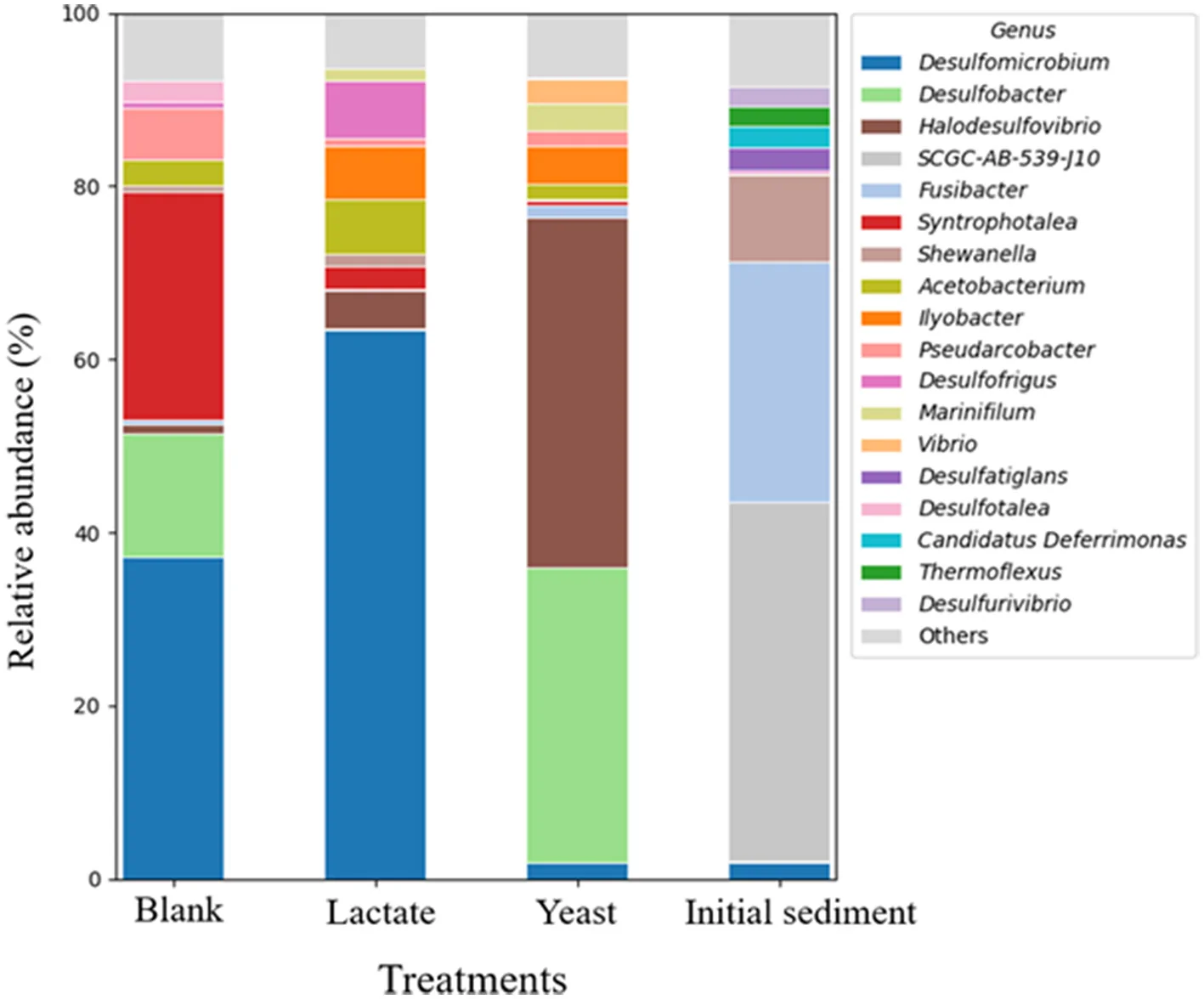
Stacked bar plot showing the relative abundance of ASVs summarized on the taxonomic level of genus after 28 days of experiment. Taxa (%) by Treatment—≥2% shown.

**Figure 11:**
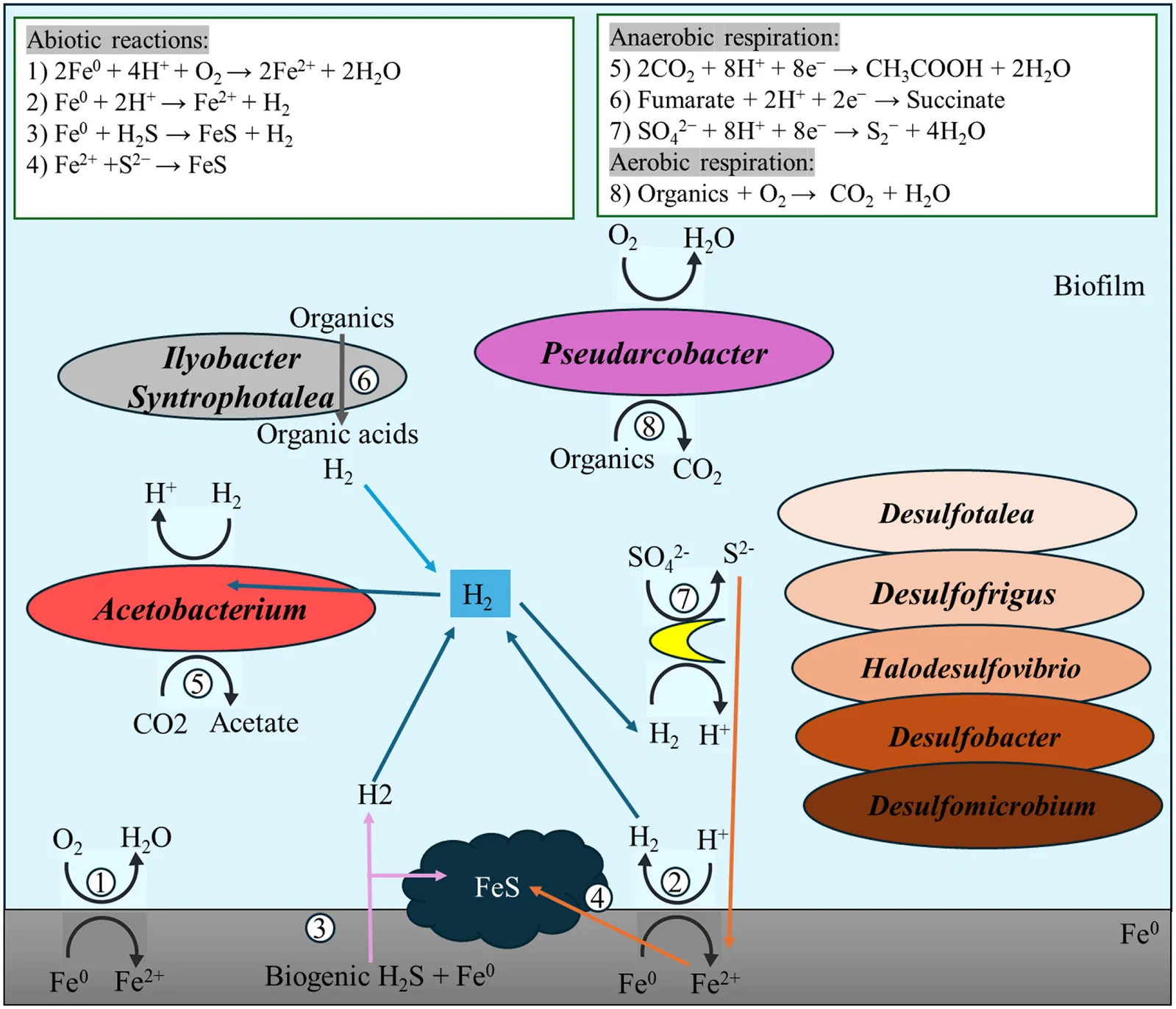
Conceptual model illustrating microbial diversity and spatial heterogeneity within the biofilm investigated in this study under different nutrient treatments, adapted from Xu et al. (2023). Equations 1–4 represent abiotic reactions associated with iron corrosion. Equations 5–7 depict anaerobic respiratory pathways carried out by the microbial community. Hydrogen (H_2_), generated from metallic iron (Fe°) and produced by fermentative taxa (gray), is consumed by sulfate-reducing bacteria (brown shades), and acetogens (red). Fe(III) reducers (orange boxes) directly extract electrons from Fe^0^ generating ferrous ion (Fe^2+^). Heterotrophic taxa (magenta) may further scavenge residual oxygen, reinforcing redox stratification and promoting anaerobic corrosion processes (Equation 8).

Figure 10 illustrates the genus-level community composition across different nutrient treatments and in the initial sediment inoculum, suggesting a distinct shifts in microbial structure associated with nutrients. A core group of genera (including *Desulfomicrobium, Acetobacterium, Pseudarcobacter*, and *Halodesulfovibrio*) was detected across all treatments, however, their relative abundances varied significantly depending on the nutrient treatment. *Desulfomicrobium* was significantly enriched in blank and lactate-amended systems compared with the yeast treatment and the initial sediment. This enrichment indicates that the incubation conditions favored sulfate-reducing populations associated with anaerobic carbon steel corrosion. Mechanistically, *Desulfomicrobium* is relevant because it can incompletely oxidize organic substrates, such as lactate, to acetate while using sulfate as a terminal electron acceptor (Kuever and Galushko, 2014). This process produces sulfide and contributes to the formation of sulfidic microenvironments at the metal–biofilm interface. Therefore, its higher enrichment in lactate-amended systems suggests a potential link between lactate utilization, sulfate reduction, sulfide production, and corrosion activity. *Acetobacterium* was also detected across treatments and was relatively abundant in blank and lactate-amended systems. Members of this genus are acetogenic bacteria capable of reducing CO_2_ to acetate via the Wood–Ljungdahl pathway. They can use substrates such as H_2_/CO_2_, formate, alcohols, and sugars, producing acetate as a central metabolic product (Ross et al., 2020). In the context of MIC, *Acetobacterium* may contribute to cross-feeding by supplying acetate or influencing H_2_/formate turnover, thereby supporting SRB such as *Desulfomicrobium* and *Halodesulfovibrio*. This suggests a possible metabolic coupling between acetogenesis, fermentation, and sulfate reduction in the biofilm community. *Halodesulfovibrio* was more pronounced in the yeast extract treatment. This genus comprises halophilic SRB adapted to marine environments. *Halodesulfovibrio* can reduce sulfate and related sulfur compounds to H_2_S while incompletely oxidizing substrates such as lactate, pyruvate, alcohols, hydrogen, or formate, often producing acetate as an end product. Its enrichment under yeast extract conditions suggests that complex organic substrates may have supported marine SRB capable of active sulfate reduction (Jafari et al., 2024). *Ilyobacter* showed higher abundance in lactate and yeast extract treatments. *Ilyobacter* species are fermentative bacteria that obtain energy through substrate-level phosphorylation and can degrade organic acids and sugars, including lactate, pyruvate, and 3-hydroxybutyrate. Their metabolism can generate acetate, H_2_, and other fermentation products, which may subsequently serve as electron donors or carbon sources for sulfate reducers (Brune et al., 2002). Thus, the presence of *Ilyobacter* indicates that fermentative substrate conversion may have contributed to the availability of low-molecular-weight metabolites supporting SRB activity. *Syntrophotalea* was enriched under blank/no-nutrient conditions, indicating the potential importance of syntrophic metabolism when no external organic carbon was supplied. *Syntrophotalea* species can degrade substrates such as alcohols, amino acids, and acetylene into acetate, H_2_, and formate. Their metabolism is strictly syntrophic and depends on partner microorganisms that consume H_2_ and formate, thereby maintaining thermodynamically favorable conditions for substrate degradation (Day et al., 2022). Under carbon-limited conditions, such syntrophic interactions may become particularly important by recycling endogenous or sediment-derived organic compounds and supplying electron donors to SRB. This may help explain why the blank treatment showed the highest corrosion and pitting rates despite not having the highest bulk sulfide concentration. Overall, the community data suggest that corrosion development was associated not with a single microbial taxon but with a network of interacting metabolic guilds. Fermentative and acetogenic bacteria, such as *Ilyobacter, Syntrophotalea*, and *Acetobacterium*, may have supplied acetate, H_2_, and formate, while SRB, including *Desulfomicrobium* and *Halodesulfovibrio*, used these substrates to drive sulfate reduction and sulfide formation. Figure 11 shows a conceptual model of microbial diversity and spatial heterogeneity within the biofilm investigated in this study under different nutrient treatments, adapted from Xu et al. (2023).

## 4 Discussion

This study shows that nutrient availability influenced microbial community structure, sulfur chemistry, and corrosion behavior in sediment-derived biofilms on carbon steel. The corrosion response under blank/no-nutrient conditions was characterized by the highest mean corrosion rate, the highest pitting rate, and a greater occurrence of large-volume pits. In contrast, lactate and yeast extract supported lower corrosion and pitting rates despite promoting microbial activity and, in the case of yeast extract, substantial sulfide accumulation. This pattern suggests that nutrient-rich conditions can enhance bulk microbial activity and sulfide production without necessarily intensifying localized metal attack. Under carbon-limited conditions, the microbial community may instead shift toward a biofilm state in which metabolism is more closely coupled to the steel surface or to internally recycled organic matter within the sediment–biofilm matrix. Such conditions may favor localized metabolic interactions, stronger chemical gradients, and potentially a greater role of direct or indirect electron acquisition from the metal surface. This interpretation is consistent with the findings of Wang et al. (2024), who showed that carbon-source starvation increased the attachment of *Desulfovibrio vulgaris* to X80 steel and promoted localized corrosion by encouraging the cells to obtain electrons from the metal. Their results further demonstrated that the addition of the electron mediator riboflavin enhanced EET and increased corrosion, showing that nutrient limitation and electron-transfer capacity can jointly regulate MIC severity (Wang et al., 2024). Although the present study involved a mixed sediment-derived community rather than a single SRB strain, the higher corrosion observed in the blank treatment is compatible with a shift from readily available organic substrates toward more surface-associated electron acquisition under carbon limitation.

The microbial community data support this interpretation. PERMANOVA confirmed that nutrient treatment significantly shaped community composition, while PERMDISP indicated that this effect was not primarily caused by unequal within-group dispersion. The genus-level profiles suggest that corrosion was associated with a network of interacting metabolic guilds rather than a single dominant taxon. SRB such as *Desulfomicrobium* and *Halodesulfovibrio* provide a direct route for sulfate reduction and sulfide formation, while fermentative, acetogenic, and syntrophic taxa such as *Ilyobacter, Acetobacterium*, and *Syntrophotalea* may contribute acetate, H_2_, formate, or other intermediates that support sulfate reduction and biofilm metabolism. The presence of such taxa under blank/no-nutrient conditions is particularly relevant because it suggests that, when external organic carbon is limited, the community may rely more strongly on internal recycling of sediment-derived or biofilm-derived substrates (Day et al., 2022).

Recent studies support the need to interpret MIC through functional guilds and microbial interactions rather than through SRB abundance or sulfide concentration alone. In sulfide-induced sewer corrosion systems, sulfur chemistry was shown to depend on interactions among SRB, SOB, fermentative organisms, amino-acid-metabolizing bacteria, and methanogens (Sudiartha and Imai, 2025). This is relevant to the present study because lactate and yeast extract likely affected corrosion not only as simple carbon additions, but also by changing the flow of fermentation products, amino-acid-derived substrates, and sulfur intermediates through the biofilm community. In particular, lactate can be converted to acetate and other low-molecular-weight intermediates, while yeast extract supplies complex organic compounds, including amino acids and peptides, that may stimulate broader heterotrophic and syntrophic metabolism. Therefore, the lower corrosion observed in the amended treatments may reflect a shift toward bulk-phase or biofilm-associated metabolism that did not necessarily promote the most aggressive localized attack at the steel surface.

The importance of localized microenvironments is also supported by field studies of macrofouling-covered steel. In those systems, organic secretions from fouling organisms supplied nutrients, while the physical coverage created local anaerobic zones at the steel interface. These conditions enriched SRB such as *Desulfovibrio* and *Desulfobulbus*, increased dsr-related sulfate-reduction potential, promoted FeS formation, and caused severe localized corrosion (Wang et al., 2023). Although the present study did not involve macrofouling, it similarly highlights that corrosion-relevant processes occur within the steel–biofilm/deposit microenvironment rather than being fully reflected by bulk aqueous measurements. The measured dissolved sulfide concentration should therefore be interpreted as an end-point bulk aqueous parameter, not as a direct measure of sulfide availability, sulfur transformations, or corrosive chemistry within pores, pits, and corrosion-product deposits. Local pH, redox potential, sulfide gradients, iron sulfide precipitation, and unmeasured sulfur species such as sulfite, thiosulfate, polysulfides, and elemental sulfur may have differed substantially from the bulk phase and contributed to the observed corrosion response. Recent work in a sea-mud system further showed that substantial changes in corrosion severity can occur without corresponding increases in planktonic or sessile cell numbers. Gai et al. (2026) found that a residual magnetic field markedly intensified SRB-induced pitting and weight loss while having little effect on microbial growth or sulfate-reduction activity when organic carbon was available. Their electrochemical and cyclic-voltammetry results indicated that the main effect was an enhancement of EET, partly associated with magnetite-containing corrosion products (Gai et al., 2026). This supports the interpretation that interfacial electron-transfer efficiency and corrosion-product properties may be more informative indicators of MIC severity than bulk microbial abundance alone.

The alpha-diversity results should also be interpreted in this context. Higher richness or Shannon diversity does not necessarily indicate a more corrosive community. Previous field-based work showed that steel surfaces without macrofouling coverage had the highest microbial richness and diversity but experienced less severe MIC than oyster-covered interfaces (Wang et al., 2023). This supports the interpretation that MIC severity is not determined by total diversity alone, but rather by the enrichment and activity of specific functional guilds and their spatial organization at the metal surface. Similarly, studies of steel corrosion in soil showed that corrosion enriched specific iron-oxidizing, nitrifying, and denitrifying taxa and strengthened pathways related to nitrogen cycling, biofilm formation, quorum sensing, and extracellular electron transfer, even though SRB were not dominant (Huang et al., 2021). Together, these studies support a broader ecological interpretation in which MIC can arise from coupled redox processes and microbial interactions, rather than from sulfide production or SRB abundance alone.

The chloride concentration of the seawater used in this study should also be considered when interpreting the results. The measured chloride concentration was 15,824 mg L^−1^, which is lower than typical open North Sea seawater and corresponds to approximately half-strength marine salinity. This difference is relevant because salinity and chloride concentration can influence SRB physiology, sulfide speciation, solution conductivity, chloride-induced depassivation, and corrosion electrochemistry. Recent work on SRB-induced MIC under different chloride concentrations showed that chloride effects are non-linear: corrosion severity did not simply increase with sulfate reduction activity, but was also linked to EPS production, Fe^2+^ complexation, and redox-active functional groups involved in EET (Li et al., 2026). Therefore, the chloride level used here represents an important experimental condition and limits direct extrapolation to fully marine offshore North Sea salinity.

## 5 Conclusion

This study investigated how nutrient availability under simulated near-seabed conditions influences microbial community structure, metabolic activity, and corrosion behavior of carbon steel. By integrating 16S rRNA sequencing, dissolved sulfide measurements, corrosion metrics, 3D surface profilometry, and untargeted metabolomics, the results show that blank, lactate, and yeast-extract treatments generated distinct microbial and chemical environments. Yeast extract treatment produced the highest total sulfide concentration, yet corrosion severity did not scale with sulfide accumulation. Instead, the highest corrosion and pitting rates were observed under carbon-limited blank conditions. These findings indicate that carbon limitation can promote localized MIC even when nutrient-amended systems show stronger bulk microbial activity or higher sulfide production. Overall, the study highlights that MIC in sediment-associated marine environments is governed by the combined effects of substrate availability, microbial community structure, metabolic interactions, and biofilm–corrosion product heterogeneity. From an engineering perspective, the results suggest that nutrient-rich laboratory media may not fully reproduce corrosion behavior under field-relevant, low-labile-carbon sediment conditions. Therefore, future testing of offshore foundation materials should include carbon-limited scenarios that better represent deeper, diffusion-limited, or refractory organic-matter-dominated sediment zones. Inspection and monitoring strategies should also give particular attention to localized attack, pit morphology, and deposit heterogeneity, since deep pitting beneath heterogeneous biofilm–corrosion product layers may be more relevant for structural integrity than average corrosion rates alone.

## 6 Limitations and future work

Several limitations should be considered when interpreting the results of this study. First, the 28-day exposure period represents a short-term laboratory screening designed to compare microbial and corrosion responses under controlled nutrient conditions. It should therefore not be interpreted as a direct simulation of long-term marine service conditions in offshore wind foundations, where exposure times, sediment consolidation, hydrodynamics, temperature fluctuations, and geochemical gradients develop over much longer timescales. Second, although weight loss and 3D optical profilometry provided quantitative information on general corrosion and localized pitting, electrochemical measurements were not included. Techniques such as open-circuit potential monitoring and electrochemical impedance spectroscopy would be needed to resolve corrosion kinetics, cathodic reactions, and possible electron-transfer processes at the biofilm–metal interface. Third, EDS analysis provided useful elemental information on sulfur enrichment within corrosion products, but it cannot identify specific mineral phases. Phase-sensitive techniques such as XRD, Raman spectroscopy, or X-ray photoelectron spectroscopy would be required to confirm whether the sulfur-rich deposits consisted of FeS, FeS_2_, greigite, pyrrhotite, or other iron sulfide phases. Future work should combine longer-term exposure experiments with electrochemical monitoring, phase-specific corrosion product characterization, transcriptomic or metatranscriptomic analysis, and field validation to deeper investigate the mechanisms suggested by the present short-term laboratory study. Although the original environmental samples are no longer available for additional analyses, the experimental design and analytical workflow are fully described and can be reproduced in future studies using newly collected seawater and sediment from the same or comparable North Sea sampling conditions, thereby enabling independent validation and extension of the present findings.

## Data Availability

The 16S rRNA sequence analysis data presented in the study are deposited in the ENA (European Nucleotide Archive) repository, accession number PRJEB123364. The metabolomics data presented in the study are deposited in the MetaboLights repository, accession number MTBLS15254.

## References

[B1] 25178-6:2010 I. (2010). Geometrical Product Specifications (GPS)—surface Texture: Areal. Part 6: Classification of Methods for Measuring Surface Texture. Geneva: ISO.

[B2] An B. A. Kleinbub S. Ozcan O. Koerdt A. (2020). Iron to gas: versatile multiport flow-column revealed extremely high corrosion potential by methanogen-induced microbiologically influenced corrosion (Mi-MIC). Front Microbiol. 11:527. doi: 10.3389/fmicb.2020.00527 32296410 PMC7136402

[B3] Bavi K. Khavari-Nejad R. A. Najafi F. Ghanati F. (2022). Phenolics and terpenoids change in response to yeast extract and chitosan elicitation in Zataria multiflora cell suspension culture. 3 Biotech 12:163. doi: 10.1007/s13205-022-03235-x PMC9271148 35822153

[B4] Brune A. Evers S. Kaim G. Ludwig W. Schink B. (2002). Ilyobacter insuetus sp. nov., a fermentative bacterium specialized in the degradation of hydroaromatic compounds. Int. J. Syst. Evol. Microbiol. 52, 429–432. doi: 10.1099/00207713-52-2-429 11931152

[B5] Callahan B. J. mcmurdie P. J. rosen M. J. Han A. W. Johnson A. J. A. Holmes S. P. (2016). DADA2: high-resolution sample inference from Illumina amplicon data. Nat. Methods 13, 581–583. doi: 10.1038/nmeth.3869 27214047 PMC4927377

[B6] Christiansen N. Daewel U. Schrum C. (2026). Cumulative hydrodynamic impacts of offshore wind farms on North Sea currents and surface temperatures. Commun. Earth Environ. 7:164. doi: 10.1038/s43247-026-03186-8

[B7] Cui Y. Pan X. Li Y. Ding Q. Lv J. (2025). Study on the effect of electronic carriers on extracellular electron transfer microbial corrosion in carbon starvation environment. Chem. Papers 80, 1037–1053. doi: 10.1007/s11696-025-04446-1

[B8] Day L. A. Kelsey E. L. Fonseca D. R. Costa K. C. (2022). Interspecies formate exchange drives syntrophic growth of Syntrophotalea carbinolica and Methanococcus maripaludis. Appl. Environ. Microbiol. 88:e01159-22. doi: 10.1128/aem.01159-22 36374033 PMC9746305

[B9] Duan Y. Sun J. (2023). Preparation of iron-based sulfides and their applications in biomedical fields. Biomimetics 8:177. doi: 10.3390/biomimetics8020177 37218763 PMC10204475

[B10] Engl T. Jakubova L. Skrob Z. Campeggi S. Skala R. Folkmanova M. . (2026). Catabolism of acetosyringone and co-metabolic transformation of 2, 4, 6-trichlorophenol by a novel FAD-dependent monooxygenase. mSystems 11:e01242-25. doi: 10.1128/msystems.01242-25 41589901 PMC12911418

[B11] Enning D. Garrelfs J. (2014). Corrosion of iron by sulfate-reducing bacteria: new views of an old problem. Appl. Environ. Microbiol. 80, 1226–1236. doi: 10.1128/AEM.02848-13 24317078 PMC3911074

[B12] Enning D. Venzlaff H. Garrelfs J. Dinh H. T. Meyer V. Mayrhofer K. . (2012). Marine sulfate-reducing bacteria cause serious corrosion of iron under electroconductive biogenic mineral crust. Environ. Microbiol. 14, 1772–87. doi: 10.1111/j.1462-2920.2012.02778.x 22616633 PMC3429863

[B13] Gai Y.-J. Li J.-H. Xie F. Wang D. Sun D.-X. Wang Y.-X. Wu M. (2026). Residual magnetic fields from magnetic flux leaking detection accelerate microbiologically influenced corrosion of X80 pipeline steel induced by Desulfovibrio desulfuricans in sea mud environment. Petrol. Sci. 23, 3602–3615. doi: 10.1016/j.petsci.2026.01.023

[B14] Grove A. (2025). The delicate balance of bacterial purine homeostasis. Discov. Bact. 2:14. doi: 10.1007/s44351-025-00025-7

[B15] Guan F. Pei Y. Duan J. Sand W. Zhang R. Zhai X. . (2024). Effect of yeast extract on microbiologically influenced corrosion of X70 pipeline steel by Desulfovibrio bizertensis SY-1. Bioelectrochemistry 157:108650. doi: 10.1016/j.bioelechem.2024.108650 38286079

[B16] Huang Y. Xu D. Huang L.-Y. Lou Y.-T. Muhadesi J.-B. Qian H.-C. . (2021). Responses of soil microbiome to steel corrosion. npj Biofilms Microb. 7:6. doi: 10.1038/s41522-020-00175-3 PMC7820017 33479252

[B17] Jafari M. Moghimi H. Tirandaz H. Ebrahim-Habibi M.-B. (2024). Corrosion behavior of predominant Halodesulfovibrio in a marine SRB consortium and its mitigation using ZnO nanoparticles. Sci. Rep. 14:19545. doi: 10.1038/s41598-024-70654-6 39174663 PMC11341846

[B18] Jørgensen B. B. Marshall I. P. (2016). Slow microbial life in the seabed. Annu. Rev. Mar. Sci. 8, 311–332. doi: 10.1146/annurev-marine-010814-015535 26209150

[B19] Kennelly C. Tran P. Prindle A. (2024). Environmental purines decrease Pseudomonas aeruginosa biofilm formation by disrupting c-di-GMP metabolism. Cell Rep. 43:114154. doi: 10.1016/j.celrep.2024.114154 38669142 PMC11197132

[B20] Kotu S. P. Wunch K. Skovhus T. L. An-Stepec B. A. Mana S. K. (eds.). (2025). “Management of microbiological challenges in the energy industry,” in Microbiological Challenges in the Energy Industries, eds. S. P. Kotu, T. L. Skovhus, and K. Wunch (Boca Raton, FL: CRC Press), 298. doi: 10.1201/9781003469643

[B21] Kotu S. P. Yang F. Klemashevich C. Sam Mannan M. Jayaraman A. (2024). “Metagenomic and metabolomic analysis of microbiologically influenced corrosion of carbon steel in produced water,” in Petroleum Microbiology, eds. S. P. Kotu, T. L. Skovhus, and K. Wunch (Boca Raton, FL: CRC Press), 96–119. doi: 10.1201/9781003287056-8

[B22] Kuever J. Galushko A. (2014). “The family desulfomicrobiaceae,” in The Prokaryotes: Deltaproteobacteria and Epsilonproteobacteria (Moscow: SciELO), 97–102 doi: 10.1007/978-3-642-39044-9_310

[B23] Li J. H. Zhou E. Z. Li Z. Wang D. Wang F. H. Xu D. K. (2026). Chloride-dependent enhancement of microbiologically influenced corrosion on X80 steel by Desulfovibrio desulfuricans: the critical role of extracellular polymeric substances. Petrol. Sci. 23, 2106–2119. doi: 10.1016/j.petsci.2025.12.004

[B24] Li Z. Yang J. Guo H. Kumseranee S. Punpruk S. Mohamed M. E. . (2021). Carbon source starvation of a sulfate-reducing bacterium–elevated MIC deterioration of tensile strength and strain of X80 pipeline steel. Front. Mater. 8:794051. doi: 10.3389/fmats.2021.794051

[B25] Liu Y. Li Q. Liu R. Wang Z. Zhao S. (2026). Exploring the multifunctional roles of betaine: traditional applications, emerging technologies, and green chemistry Innovations. Foods 15:737. doi: 10.3390/foods15040737 41750929 PMC12939989

[B26] Martin M. (2011). Cutadapt removes adapter sequences from high-throughput sequencing reads. EMBnet J. 17, 10–12. doi: 10.14806/ej.17.1.200

[B27] McMurdie P. J. Holmes S. (2013). phyloseq: an R package for reproducible interactive analysis and graphics of microbiome census data. PLoS ONE 8:e6121. doi: 10.1371/journal.pone.0061217 PMC3632530 23630581

[B28] Meng G. Z. Zhang C. Cheng Y. F. (2008). Effects of corrosion product deposit on the subsequent cathodic and anodic reactions of X-70 steel in near-neutral pH solution. Corros. Sci. 50, 3116–3122. doi: 10.1016/j.corsci.2008.08.026

[B29] Mielke R. E. Robinson K. J. White L. M. McGlynn S. E. McEachern K. Bhartia R. . (2011). Iron-sulfide-bearing chimneys as potential catalytic energy traps at life's emergence. Astrobiology 11, 933–950. doi: 10.1089/ast.2011.0667 22111762

[B30] Momber A. (2024). Corrosion and Corrosion Protection of Wind Power Structures in Marine Environments: Volume 1: Introduction and Corrosive Loads. Cambridge, MA: Academic Press. doi: 10.1016/B978-0-323-85742-0.00001-9

[B31] Nagy P. Pálinkás Z. Nagy A. Budai B. Tóth I. Vasas A. (2014). Chemical aspects of hydrogen sulfide measurements in physiological samples. Biochim. Biophys. Acta Gener. Sub. 1840, 876–891. doi: 10.1016/j.bbagen.2013.05.037 23769856

[B32] Pu Y. Tian Y. Hou S. Dou W. Chen S. (2023). Carbon starvation considerably accelerated nickel corrosion by Desulfovibrio vulgaris. Bioelectrochemistry 153:108453. doi: 10.1016/j.bioelechem.2023.108453 37230047

[B33] Quast C. Pruesse E. Yilmaz P. Gerken J. Schweer T. Yarza P. . (2012). The SILVA ribosomal RNA gene database project: improved data processing and web-based tools. Nucl. Acids Res. 41, D590–D596. doi: 10.1093/nar/gks1219 23193283 PMC3531112

[B34] Reese B. K. Finneran D. W. Mills H. J. Morse J. W. (2011). Examination and refinement of the determination of aqueous hydrogen sulfide by the methylene blue method. Aquat. Geochem. 17, 567–582. doi: 10.1007/s10498-011-9128-1

[B35] Rivera-Alvarez I. Brown R. K. Keskin-Pyttel D. Steffens J. Farber P. Schröder U. (2020). Correlating theoretical boundary layer thickness to the power output of a microbial fuel cell with a complex anode geometry operated at varying flow rates. J. Power Sources 470:228428. doi: 10.1016/j.jpowsour.2020.228428

[B36] Ross D. E. Marshall C. W. Gulliver D. May H. D. Norman R. S. (2020). Defining genomic and predicted metabolic features of the Acetobacterium genus. Msystems 5, 10–1128. doi: 10.1128/mSystems.00277-20 PMC7498680 32934112

[B37] Salazar N. González S. de los Reyes Gavilan C. G. Rios-Covian D. (2022). “Branched short-chain fatty acids as biological indicators of microbiota health and links with anthropometry,” in Biomarkers in Nutrition, eds., V. B. Patel, and V. R. Preedy (Cham: Springer), 1–17. doi: 10.1007/978-3-030-81304-8_4-1

[B38] Schymanski E. L. Jeon J. Gulde R. Fenner K. Ruff M. Singer H. P. & Hollender J. (2014). Identifying small molecules via high resolution mass spectrometry: communicating confidence. Environ. Sci. Technol. 48, 2097–2098. doi: 10.1021/es5002105 24476540

[B39] Shojai S. Schönamsgruber F. Koehler M. Ghafoori E. (2025). Impact of accelerated corrosion on weld geometry, hardness and residual stresses of offshore steel joints over time. Mater. Design 251:113578. doi: 10.1016/j.matdes.2024.113578

[B40] SP0775-2013 N. S. (2013). Preparation, Installation*, Analysis, and Interpretation of Corrosion Coupons in Oilfield Operations*. Mumbai: NACE International.

[B41] Stein J. L. Chaturvedi T. Skovhus T. L. Thomsen M. H. (2025). Applicability of yeast extract in postgate culture medium for microbiologically influenced corrosion tests. Corrosion 81, 48–57. doi: 10.5006/4609

[B42] Subramoni S. Nathoo N. Klimov E. Yuan Z. C. (2014). Agrobacterium tumefaciens responses to plant-derived signaling molecules. Front. Plant Sci. 5:322. doi: 10.3389/fpls.2014.00322 25071805 PMC4086400

[B43] Sudiartha G. A. W. Imai T. (2025). Harnessing conductive materials to reshape sewer microbiomes and mitigate corrosion from sulfide and hydrogen sulfide formation. Sci. Rep. 15:21382. doi: 10.1038/s41598-025-06099-2 40593098 PMC12216032

[B44] Tsugawa H. Cajka T. Kind T. Ma Y. Higgins B. Ikeda K. . (2015). MS-DIAL: data-independent MS/MS deconvolution for comprehensive metabolome analysis. Nat. Methods 12, 523–526. doi: 10.1038/nmeth.3393 25938372 PMC4449330

[B45] Tüccar T. Knisz J. Eckert R. Skovhus T. L. (2025). Ontology study: harmonizing microbiologically influenced corrosion (MIC) terminology across disciplines. npj Mater. Degrad. 10:6. doi: 10.1038/s41529-025-00716-1

[B46] Tung T. T. Jakobsen T. H. Dao T. T. Fuglsang A. T. Givskov M. Christensen S. B. . (2017). Fusaric acid and analogues as Gram-negative bacterial quorum sensing inhibitors. Eur. J. Med. Chem. 126, 1011–1020. doi: 10.1016/j.ejmech.2016.11.044 28033578

[B47] Wang Q. Wang B. Zhou X. Tan Z. Zhang M. Luo J. . (2024). Effects of carbon source starvation and riboflavin addition on selective corrosion of welded joint by Desulfovibrio vulgaris. Corros. Sci. 230:111931. doi: 10.1016/j.corsci.2024.111931

[B48] Wang Y. Guo D. Zhang R. Liu J. Zhang Y. Etim I. I. N. . (2026). Metabolic shift and enhanced electron transfer in sulfate-reducing bacteria induced by corrosion-biomineralized iron sulfide nanoparticles. Bioelectrochemistry 171:109295. doi: 10.1016/j.bioelechem.2026.109295 41946215

[B49] Wang Z. Wang X. Huang Y. Hou B. (2023). Metagenomic insights into nutrient and hypoxic microbial communities at the macrofouling/steel interface leading to severe MIC. npj Mater. Degrad. 7:41. doi: 10.1038/s41529-023-00365-2

[B50] Wickham H. (2016). “Data analysis,” in ggplot2: Elegant Graphics for Data Analysis (Cham: Springer), 189–201. doi: 10.1007/978-3-319-24277-4_9

[B51] Xu D. Gu T. (2014). Carbon source starvation triggered more aggressive corrosion against carbon steel by the Desulfovibrio vulgaris biofilm. Int. Biodeterior. Biodegrad. 91, 74–81. doi: 10.1016/j.ibiod.2014.03.014

[B52] Xu D. Gu T. Lovley D. R. (2023). Microbially mediated metal corrosion. Nat. Rev. Microbiol. 21, 705–718. doi: 10.1038/s41579-023-00920-3 37344552

[B53] Yang Y. Wang Y. Du X. Lin T. Wang H. Meng F. . (2024). Influence of ultraviolet light and alternating wet–dry environments on the corrosion behavior of weathering steels. Materials 17, 3870. doi: 10.3390/ma17153870 39124534 PMC11313933

[B54] Yuxuan Z. Jiaqi H. Li Z. Jin Z. Haixian L. Liu L. . (2022). Corrosion of aluminum alloy 7075 induced by marine Aspergillus terreus with continued organic carbon starvation. NPJ Mater. Degrad. 6:27. doi: 10.1038/s41529-022-00236-2

