# Supplementary material for: Carbon starvation enhances microbiologically influenced corrosion in marine offshore infrastructures
Source: Front Microbiol. 2026 Aug 31;17:1908395. doi: 10.3389/fmicb.2026.1908395 (PMC13572702; doi:10.3389/fmicb.2026.1908395)
Supplement: Supplementary file 1 [file Supplementary_file_1.docx]

Supplementary materials

**Carbon starvation enhances microbiologically influenced corrosion in marine offshore infrastructures**

Sara Taghavi Kalajahi^1*^, Jan Lisec^1^, Elyas Ghafoori^2^, Maria Salta^3,4^, Torben Lund Skovhus^5^, Andrea Koerdt^1*^

1- Federal Institute for Materials Research and Testing (BAM), Berlin, Germany.
2- Institute for Steel Construction, ForWind, Leibniz University Hannover (LUH), Hannover, Germany
3- Endures B.V. (Department: Biofilms, Biofouling and Fouling Control), Den Helder, The Netherlands.
4- School of the Environment and Life Sciences, University of Portsmouth, Portsmouth, England.
5- VIA University College, Banegårdsgade 2, 8700 Horsens, Denmark.

**Corresponding author:** Sara Taghavi Kalajahi ([sara.taghavi-kalajahi@bam.de](mailto:sara.taghavi-kalajahi@bam.de)) and Andrea Koerdt ([andrea.koerdt@bam.de](mailto:andrea.koerdt@bam.de))

Figure S1. Untargeted metabolomic profiles of bulk influent and effluent under different nutrient treatments. Mean peak intensities were calculated from biological replicates for each nutrient and expressed relative to the corresponding values in lactate sample. To focus on biologically relevant features, only compound peaks exhibiting intensities at least 100-fold higher than those detected in sterile control were retained for analysis. In the control samples, signal intensities were predominantly zero or near zero.

Figure S2. Alpha diversity of microbial communities across the three nutrient treatments. Three alpha diversity indices (Observed richness, Chao1 richness estimator, and Shannon diversity index) are shown for the blank, lactate, and yeast treatments.
